# Effects of Defined Pig Microbiota on Acute Salmonellosis in Gnotobiotic Piglets

**DOI:** 10.1096/fj.202503234R

**Published:** 2025-12-31

**Authors:** Igor Splichal, Katerina Polakova, Marek Sinkora, Vera Neuzil Bunesova, Nikol Modrackova, Eva Vlkova, Zdislava Kindlova, Kristyna Horvathova, Alla Splichalova

**Affiliations:** ^1^ Laboratory of Gnotobiology, Institute of Microbiology Czech Academy of Sciences Novy Hradek Czechia; ^2^ Department of Cell Biology, Faculty of Science Charles University Prague Czechia; ^3^ Department of Microbiology, Nutrition and Dietetics, Faculty of Agrobiology, Food and Natural Resources Czech University of Life Sciences Prague Prague Czechia

**Keywords:** defined pig microbiota, gnotobiotic piglet, high mobility group box 1, inflammatory cytokines, lipopolysaccharide, *Salmonella* Typhimurium, salmonellosis, Toll‐like receptors

## Abstract

*Salmonella enterica* subsp. *enterica* serovar Typhimurium (*S*. Typhimurium) is a ubiquitous *Salmonella* serovar that causes enterocolitis in humans, livestock, poultry, and some pets. Gnotobiotic animals are suitable for studying the development of initial colonization in the gastrointestinal tract, host‐microbiota cross‐talk, and interactions among microbiota strains. To clarify the impact of pre‐colonization of the germ‐free piglets with defined pig microbiota (DPM) on the subsequent infection with *S*. Typhimurium LT2 (LT2), we focused on the acute host immune response. Colonization with DPM did not cause signs of enterocolitis (sleepiness, anorexia, fever, and diarrhea), histological changes in the intestine, the density of acid mucin‐producing cells, expression of villin, claudin‐1, occludin, TLR4, MD‐2, CD14, LBP, MyD88, and TRIF, induction of inflammatory cytokines IL‐1*β*, IL‐6, IL‐8, IL‐10, IL‐12/23p40, TNF‐*α*, IFN‐*γ*, and HMGB1. LT2 translocated into the mesenteric lymph nodes, liver, spleen, and blood of the LT2 group, as well as damaged the intestinal structure, and upregulated intestinal inflammatory cytokine levels. The pre‐colonization with DPM ameliorated the LT2‐induced signs of enterocolitis, intestinal damage, and inflammatory changes. Changes in intestinal levels of soluble TLR4 and TLR2 indicate the possible inclusion of soluble TLRs in the regulation of the inflammatory process. The controlled initial colonization of the newborn gastrointestinal tract with a defined microbial consortium can increase newborn resistance to enteric pathogens and promote thriving of young animals.

## Graphical abstract

Germ‐free piglets lack protective microbiota and are highly vulnerable to *Salmonella* Typhimurium LT2 infection. Pre‐colonization with defined pig microbiota supports early immune response and intestinal barrier maturation. By measuring pathogen spread, intestinal structure, goblet cell density, cytokines, and tight‐junction markers with molecular and histological assays, the study shows that early microbiota establishment enhances host defenses and reduces disease severity. The figure was created with BioRender.com (RRID:SCR_018361).

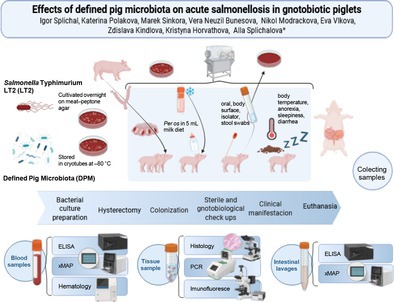

**Abbreviations**
CD14: cluster of differentiation 14
DAMP: Damage‐Associated Molecular Patterns
DPBS: Dulbecco's Phosphate Buffered Saline
DPM: defined pig microbiota
HMGB1: high mobility group box 1
IFN: interferon
IL: interleukin
LBP: lipopolysaccharide‐binding protein
LPS: lipopolysaccharide
LT2: LT2 strain of *S*. Typhimurium
MD‐2: myeloid differentiation factor 2
MLN: mesenteric lymph nodes
MyD88: myeloid differentiation primary response 88
PAMP: Pathogen‐Associated Molecular Patterns
RT: room temperature
TLR: Toll‐like receptor
TNF: tumor necrosis factor
TRIF: Toll, interleukin‐1 receptor, and resistance protein domain‐containing adaptor inducing interferon‐*β*

## 1 Introduction

Higher organisms (hosts) phylogenetically developed in the environment with the presence of simple microorganisms that colonized them. This evolutionary coexistence led to the establishment of complex symbiotic relationships (cross‐talk) between the hosts and their microbial colonizers (microbiota) [1]. The microbiota‐host relationships range from both sides of beneficial mutualism to host‐detrimental microbial parasitism [2].

Microbiota comprises myriads of microorganisms, including bacteria, archaea, fungi, algae, viruses, protists, and parasitic worms, and reaches its highest density in the host's lower gastrointestinal (GI) tract [3]. Human and other mammalian fetuses develop in the sterile conditions of the uterus [4]. The initial microbial colonization of the newborn GI tract with the mother's vaginal microbiota occurs already during birth. The early establishment of a balanced microbiota (eubiosis) is a fundamental assumption of a host's health and prosperity [5]. Cesarean section (CS) can be performed for various indications in humans [6] and also in animals, such as livestock [7] and pets [8]. The CS‐delivered newborn GI tract colonization influences environmental microbes, for example, those from hospital surroundings, as described in humans [9]. Unfortunately, it significantly increases the risk of primary seeding with multidrug‐resistant bacteria, which can be the source of nosocomial infections [10]. The need to ensure CS‐newborn health prosperity and decrease the probability of colonization with multidrug‐resistant microbes suggests a controlled primary seeding of the GI tract with host‐beneficial microbiota [11]. Such willful colonization can support the successful development of newborn hosts [1] and enhance their resistance to enteric infections [12].

*Salmonella* species are human and animal aerotolerant, flagellated, Gram‐negative bacterial pathogens. A ubiquitous *Salmonella enterica* subsp. *enterica* serovar Typhimurium (*S*. Typhimurium) belongs to the non‐typhoidal *Salmonella* serovars. It causes enterocolitis (salmonellosis) in humans and certain animals, such as pigs [13]. *S*. Typhimurium occurrence in pig herds is high; however, pigs often exhibit no apparent signs of infection (diarrhea, fever, abdominal cramps, anorexia, and sleepiness). These seemingly healthy pigs can be carriers of an infection that excretes *Salmonella* and infects other pigs [14, 15]. Infection with *Salmonella enterica* serovars decreases the economic effectiveness of the pig industry, and preventing salmonellosis is therefore necessary.

The immune system recognizes molecular structures that signal danger for the host organism. These structures are the host body's foreign Pathogen‐Associated Molecular Patterns (PAMPs), for example, lipoteichoic acid (LTA) of Gram‐positive bacteria and lipopolysaccharide (LPS) of Gram‐negative bacteria [16], and the host body's molecular structures—Damage‐Associated Molecular Patterns (DAMPs) that are normally hidden from immune recognition and that appear after tissue damage, for example, high mobility group box 1 (HMGB1) [17]. Toll‐like receptors (TLRs) are one of the five main groups of Pattern Recognition Receptors (PRRs), recognizing molecular patterns as homodimers, such as TLR4, or heterodimers, like TLR2. TLRs sense both PAMPs and DAMPs. Their signaling results in the production of inflammatory cytokines or interferons [16, 17]. The TLR4 signaling pathway is depicted in the simplified schema (Figure 14). TLRs use two adaptor proteins in their signaling pathways: MyD88 (myeloid differentiation primary response 88) and TRIF (Toll, interleukin‐1 receptor, and resistance protein domain‐containing adaptor inducing interferon‐*β*). TLR4 is the only TLR that utilizes both MyD88 and TRIF adaptor proteins [18].

*S*. Typhimurium LT2 is “a laboratory” strain of *S*. Typhimurium [19]. It caused mild diarrhea in one‐week‐old conventional piglets [20]. In contrast, the infection of one‐week‐old germ‐free (GF) piglets had lethal consequences for the piglets [21]. Thus, we prepared a defined pig microbiota (DPM) from pig commensals with in vitro–verified anti‐*Salmonella* properties [22], which is expected to suppress the multiplication of *S*. Typhimurium LT2 in the intestines of GN piglets [23].

We aimed to evaluate the effect of DPM on the activation of the innate immune response during infection with *S*. Typhimurium LT2 in one‐week‐old gnotobiotic (GN) piglets that were pre‐colonized with DPM and subsequently infected with *S*. Typhimurium LT2 for 24 h.

## 2 Materials and Methods

### 2.1 Bacterial Cultures

The DPM consortium containing nine bacterial species/strains (*Bacillus* sp., *Bifidobacterium animalis* subsp. *lactis*, *B. porcinum*, *Clostridium sporogenes* , *Lactobacillus amylovorus* , *L. paracasei* subsp. *tolerans*, *Limosilactobacillus reuteri* subsp. *suis*, and two strains of *Limosilactobacillus reuteri* subsp. *porcinus*) with expected anti‐*Salmonella* and probiotic properties was prepared as described elsewhere [22]. Briefly, individual strains were tested on anti‐*S*. Typhimurium LT2 activity using culture supernatants. Bacteria were verified for their autoaggregation, survival under low‐pH and bile‐salt conditions, antibiotic resistance, hemolytic and lecithinase activities, and adhesion to the porcine IPEC‐J2 epithelial cell line. Finally, mutual inhibition among the selected strains and the stability of the bacterial mixture were examined. The DPM was stored in cryotubes at −80°C until use, but no more than 1 month. Bacterial viability was verified before use by parallel cultivation with the in vivo experiment. Before GF piglet colonization, the DPM was moved from the freezer to a gnotobiotic (GN) isolator entry port and melted at room temperature during a 30‐min exposure of hermetically closed cryotubes to peracetic acid aerosol.

*S*. Typhimurium LT2 (LT2) was originally kindly donated by Dr. O. Lüderitz (Institute for Immunology, Freiburg Breisgau, Germany) [24]. It was maintained in the collection of microorganisms at the Laboratory of Gnotobiology of the Institute of Microbiology, Czech Academy of Sciences (Novy Hradek, Czechia). *Salmonella* was cultivated overnight on meat‐peptone agar slopes (blood agar base; Oxoid, Basingstoke, UK) and resuspended to 1 × 10^8^ CFU/mL. The number of CFUs was estimated via spectrophotometry at 600 nm and subsequently verified by cultivation methods.

### 2.2 Miniature Gnotobiotic Piglets

**FIGURE 1:**
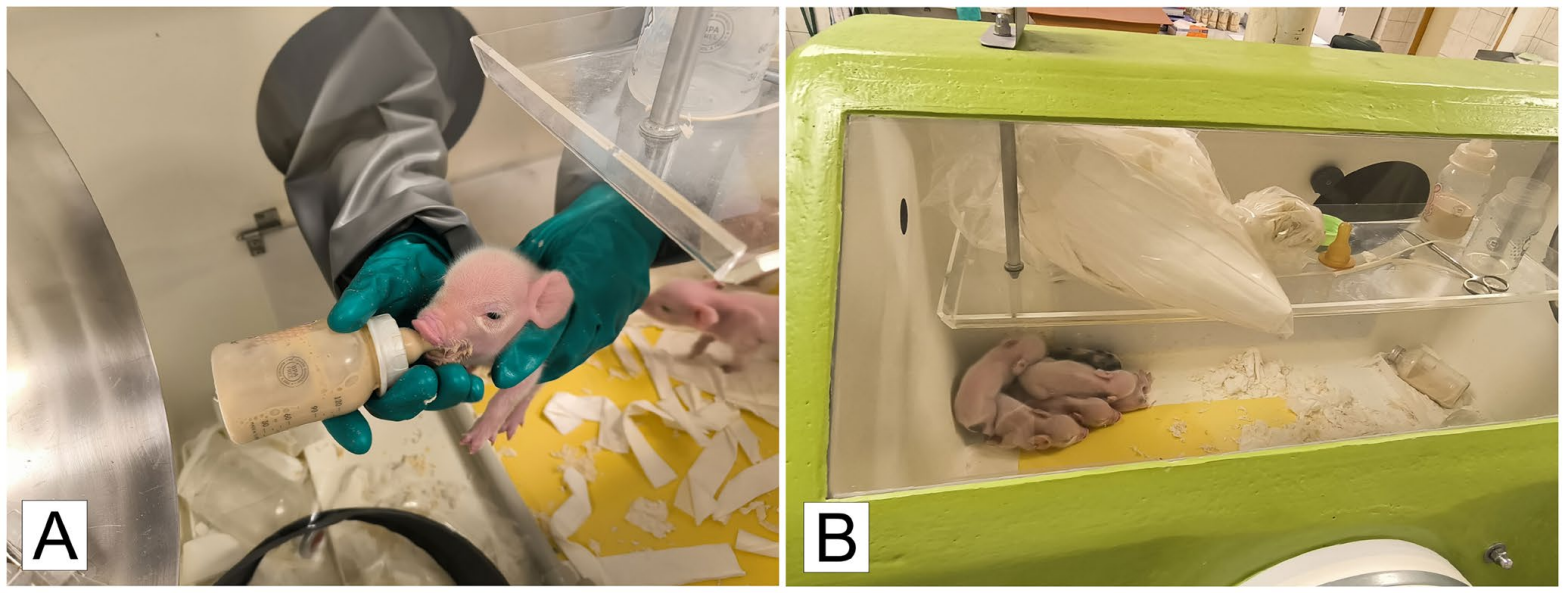
GN piglets reared in the fiberglass isolator. (A) The GN piglets were fed by a cow‐milk‐based formula by bottle with a nipple. (B) An isolator floor was partially heated to allow the piglets to choose their temperature optimum [25].

Miniature GF piglets ( *Sus scrofa* , RRID:NCBITaxon_9823) were derived by hysterectomy on expected full‐term pregnancy (112th day in the miniature pigs), bred in fiberglass gnotobiotic isolators, and fed by an autoclaved cow milk‐based diet as described elsewhere [25] (Figure 1).

**FIGURE 2:**
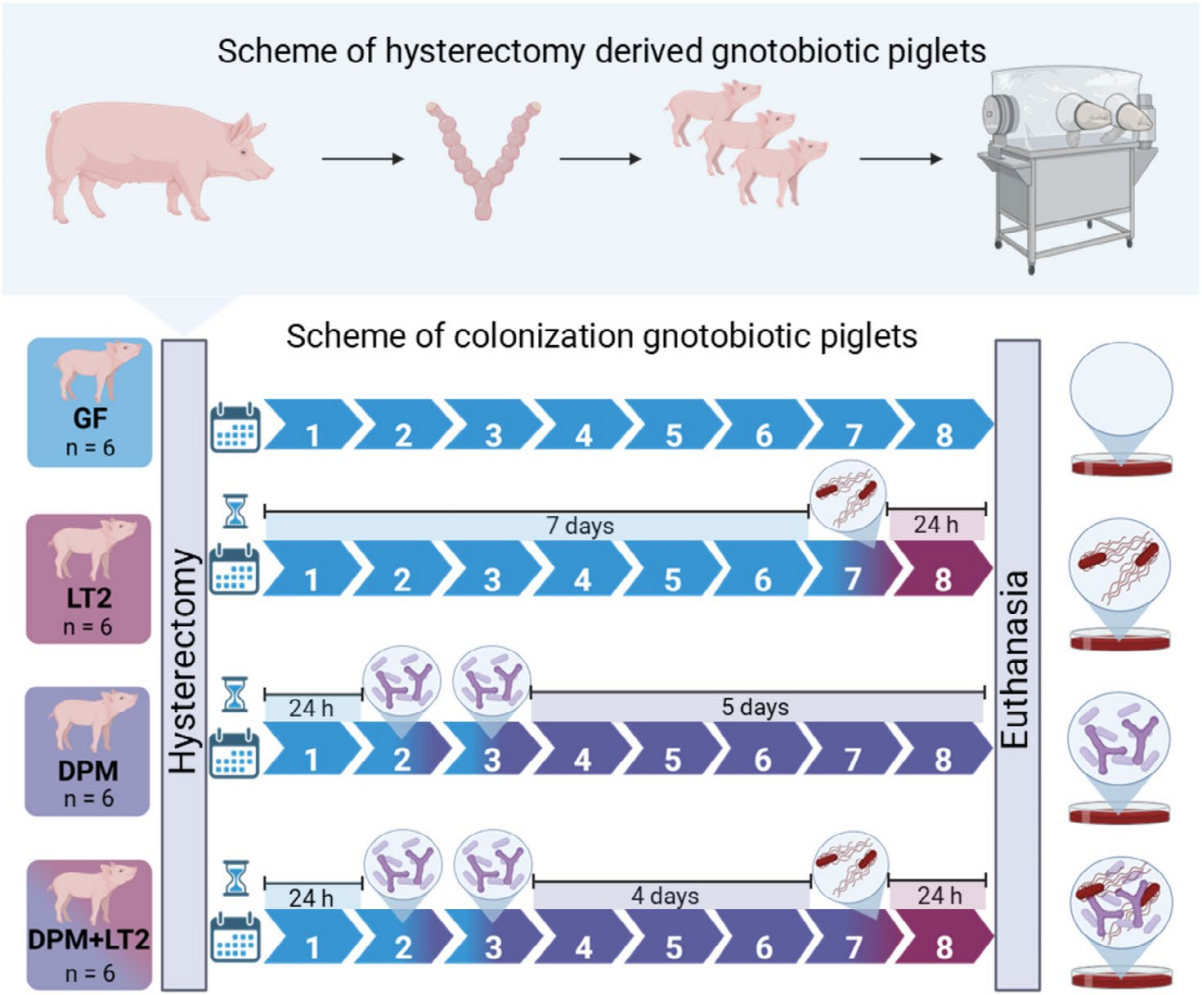
GN piglet groups. The GN piglets (*n* = 24) were assigned into four groups with six piglets per group: (i) Germ‐free (GF), (ii) infected with *S*. Typhimurium strain LT2 for 24 h (LT2), (iii) colonized with DPM (DPM), and (iv) DPM infected with *S*. Typhimurium for 24 h (DPM + LT2). The figure was created with BioRender.com (RRID:SCR_018361).

Twenty‐four piglets were randomly divided into four groups, and each piglet group consisted of six piglets from three hysterectomies (Figure 2): (i) GF for the entire eight‐day experiment (GF), (ii) seven‐day‐old GF piglets infected for 24 h with LT2 (LT2), (iii) piglets repeatedly colonized with DPM at 24 and 48 h after hysterectomy (DPM), (iv) seven‐day‐old DPM piglets infected with *S*. Typhimurium for 24 h (DPM + LT2). The bacterial inocula (LT2–1 × 10^6^ CFU, DPM 6 × 10^8^ per piglet) were orally applied in a 5 mL milk diet, and the GF group received a milk diet with cryoprotective medium without bacteria.

Sterility of the surgery and GF piglets was verified by cultivation of specimens taken at hysterectomy (amniotic membranes, umbilical cords, meconium, mouth, and isolator surface smears) for the presence of aerobic and anaerobic bacteria and molds. Later, smears from the mouth, surface of the body, and isolator, as well as stool, were tested twice a week. Additionally, Gram‐stained rectal swabs were inspected under a light microscope. The microbiota analyses of the DPM‐colonized piglets and the sterility in the GF piglets were assessed at the end of the experiments by molecular‐genetic methods, as we described elsewhere [23].

### 2.3 Clinical Signs of Salmonellosis

The GN piglets were examined for fever, anorexia, sleepiness, and diarrhea during each feeding. Diarrhea was assessed using a stool consistency score, with four degrees: 0 = normal, 1 = soft, 2 = thick, and 3 = completely liquid [26].

### 2.4 Blood Plasma

Blood collected in K3‐EDTA‐containing tubes (Cat# 01.1605.001, Sarstedt, Nümbrecht, Germany) was centrifuged at 1200 × g for 10 min at 8°C. A protease inhibitor cocktail (Cat# 04693159001, Roche Diagnostics, Mannheim, Germany) was added to the collected plasma, which was then aliquoted and stored at −40°C until further processing.

### 2.5 Intestinal Lavages

Forty centimeter of the small intestine (the whole ileum and distal part of the jejunum) were filled with 2 mL of Dulbecco's phosphate‐buffered saline (DPBS; Cat# 14190–094, Life Technologies, Carlsbad, CA, USA), mildly massaged, and flushed. The piglet's colon forms a spiral, with its ascending and descending parts fused, making it difficult to separate them to facilitate flushing. Therefore, the colon was cut into small pieces in a 90 mm Petri dish and rinsed with 4 mL of DPBS to wash its contents and mucosal part. Both types of lavages were centrifuged at 1500 × g for 30 min at 8°C. The supernatants were filtered through a 0.2 μm pore acetate cellulose syringe filter (Cat# 83.1826.102, Sarstedt), supplemented with protein inhibitors (Cat# 04693159001, Roche Diagnostics), divided into 300 μL aliquots, and stored at −40°C until the subsequent analyses [27].

### 2.6 Goblet Cell Density in the Intestine

Five μm cross‐sections of paraformaldehyde‐fixed (Cat# P6148, Sigma‐Aldrich), ethanol‐dehydrated, and paraffin‐embedded (Cat# R788272, Leica Microsystems, Wetzlar, Germany) terminal ileum and transverse colon were stained with Alcian Blue (Cat# C0052, Diapath, Martinengo, Italy) and subsequently stained with Nuclear Fast Red (Cat# C0482, Diapath). The acidic mucin‐producing goblet cells were analyzed using an Olympus BX40 microscope with an Olympus Camedia C‐2000 digital camera (Olympus, Tokyo, Japan).

### 2.7 Intestinal And Plasma Lipopolysaccharide (LPS) Concentrations

LPS detection was performed using the PyroGene Recombinant Factor C (rFC) Assay (Cat# 50‐658 U, Lonza, Basel, Switzerland). The rFC enzyme and fluorogenic substrate were added to the wells containing samples and standards, which were pre‐incubated at 37°C for 10 min. Fluorescence intensity was measured initially, followed by further incubation at 37°C for 1 h. A second fluorescence reading was then taken. All measurements were conducted using the Infinite M200 microplate reader (Tecan, Männedorf, Switzerland) with an excitation wavelength of 380 nm and an emission wavelength of 440 nm. The entire procedure was performed using LPS‐free, pyrogen‐free equipment.

### 2.8 mRNA Reverse Transcription

Cross‐section slices of the terminal ileum or transverse colon were cut and stored in RNlater (Cat# R0901, Sigma‐Aldrich, St. Louis, MO, USA) at −20°C till the following total RNA purification. RNeasy Plus Mini kit (Cat# 74134 Qiagen, Hilden, Germany) with an antifoaming reagent DX (Cat# 19088, Qiagen) was used according to the manufacturer's protocol to purify total RNA from tissues homogenized in TissueLyser LT bead beater (Qiagen) at 50 Hz for 5 min at RT with 2 mm zirconia beads (Cat# 11079124zx, BioSpec Products, Bartlesville, OK). The quantity and purity were assessed by measuring the absorbance at 260, 280, and 320 nm using a Nanodrop 1000 (Thermo Fisher Scientific, Waltham, MA, USA). Five hundred nanograms were reverse‐transcribed with a mixture of random hexamers and oligo (dT) primers in a 20 μL reaction mixture using the QuantiTect Reverse Transcription kit (Cat# 205313, Qiagen) according to the manufacturer's instructions. The PCR template was prepared by adding 180 μL of PCR‐quality water (Cat# 10977–035, Life Technologies) and stored at −25°C until the Real‐Time PCR was performed.

### 2.9 Real‐Time PCR

A total of 1.2 μL of the PCR template was added to 10.8 μL of the qPCRBIO SyGreen Blue Mix (Cat# PB20.17–51, PCR Biosystems, London, UK) containing 500 nM each of the forward and reverse primers (Generi‐Biotech, Hradec Kralove, Czech Republic) [28]. Two minutes of initial heating at 95°C was followed by 40 cycles at 95°C for 5 s and 60°C for 30 s. The mixtures were incubated, measured, and analyzed in duplicate on a qTOWER^3^ G Touch cycler and qPCRsoft 4.1 software (Analytik Jena, Jena, Germany, RRID:SCR_021910). Ct for villin, claudin‐1, occludin, TLR2, TLR4, MD‐2, LBP, CD14, MyD88, and TRIF were normalized to *β*‐actin, and their relative mRNA fold change expressions were calculated by the 2^−ΔC^_T_ method [29] by GenEx 6.1 software (MultiD Analyses AB, Gothenburg, Sweden). The used primer sequences were published elsewhere [21, 27].

### 2.10 Immunofluorescence Detection of Villin

Colon sections embedded in Tissue‐Tek OCT (Cat# 4583, Sakura Finetek, Tokyo, Japan) were snap‐frozen in liquid nitrogen vapor‐cooled isopentane and stored at −70°C. Acetone‐fixed cryosections (5 μm) were cut on a CM1860 UV cryostat (Leica Microsystems, Wetzlar, Germany), placed on SuperFrost/Plus slides (Cat# J1800AMNZ, Thermo Fisher Scientific), and stored at −40°C until labeling. Later, the sections were blocked with 5% normal goat serum (Cat# 50197Z, Thermo Fisher Scientific) for 1 h at RT, incubated with anti‐villin rabbit polyclonal antibodies (Cat# NBP1‐32841, RRID:AB_10003997, Novus Biologicals, Centennial, CO, USA) overnight at 4°C, and labeled with Alexa Fluor 488‐conjugated goat anti‐rabbit IgG antibodies (Cat# A‐11070, RRID:AB_142134, Life Technologies) for 2 h at RT. After embedding in ProLong Gold Antifade Reagent (Cat# P36941, Life Technologies), the sections were evaluated under a Leica DMi8 fluorescence microscope equipped with the THUNDER imaging platform (Leica Microsystems, Wetzlar, Germany). Acquired data were processed using LAS X software (Leica Microsystems, RRID:SCR_013673). The sections without primary antibodies served as controls.

### 2.11 ELISA

Concentrations of soluble TLR4, TLR2, and CD14 in intestinal lavages and HMGB1 in the lavages and plasma were measured using appropriate ELISA kits (Cat# abx585249, abx255571, abx154901, and abx154938, Abbexa, Cambridge, UK) according to the manufacturer's instructions. The results were measured at 450 and 620 nm in an Infinite M200 ELISA reader (Tecan) and analyzed using Magellan 6 software (Tecan, RRID:SCR_019033).

### 2.12 xMAP Technology (Luminex)

IL‐1*β*, IL‐6, IL‐8, IL‐10, IL‐12/23p40, TNF‐*α*, and IFN‐*γ* were measured in intestinal lavages and plasma using Luminex paramagnetic bead‐based xMAP technology with the Porcine Procarta Assay (Cat# EPX090‐60829‐901, Life Technologies). The analytes were measured on a Bio‐Plex multiarray system (Bio‐Rad, Hercules, TX, USA) and analyzed with Bio‐Plex Manager 4.01 (Bio‐Rad, RRID:SCR_014330).

### 2.13 Statistics

Values with a normal distribution were evaluated using a one‐way analysis of variance (ANOVA) with Tukey's multiple comparisons *post hoc* test. Values that did not have a normal distribution (Shapiro–Wilk test) were compared using the Kruskal–Wallis test, followed by Dunn's multiple comparisons *post hoc* test. Statistical comparisons were performed at *p* < 0.05 using GraphPad Prism 6 software (GraphPad Software, San Diego, CA, USA, RRID:SCR_002798), and differences among groups were denoted in the figures using a letter system (a,b,c). Student's t‐test was used for comparison of *Salmonella* CFU/mL or CFU/g, and significant differences with *p* < 0.05 were denoted by an asterisk (*).

## 3 Results

### 3.1 Clinical Signs of Salmonellosis

The GF piglets thrived for the whole experimental period. Their stool consistency was normal (score 0). Piglets infected with LT2 expressed signs of salmonellosis beginning at 6 h post‐infection. They showed anorexia, fever, sleepiness, and diarrhea (score 3). The piglets colonized with DPM were temporarily less active after colonization, but their activity later became comparable to that of the GF piglets. The stool consistency was softer than that of the GF piglets (score 1). The piglets previously colonized with DPM microbiota and later infected with LT2 showed ameliorated signs of salmonellosis, and their stool consistency score was assessed at 2.

### 3.2 *S*. Typhimurium LT2 Colony‐Forming Units in the Intestine and *Salmonella* Translocation

**FIGURE 3:**
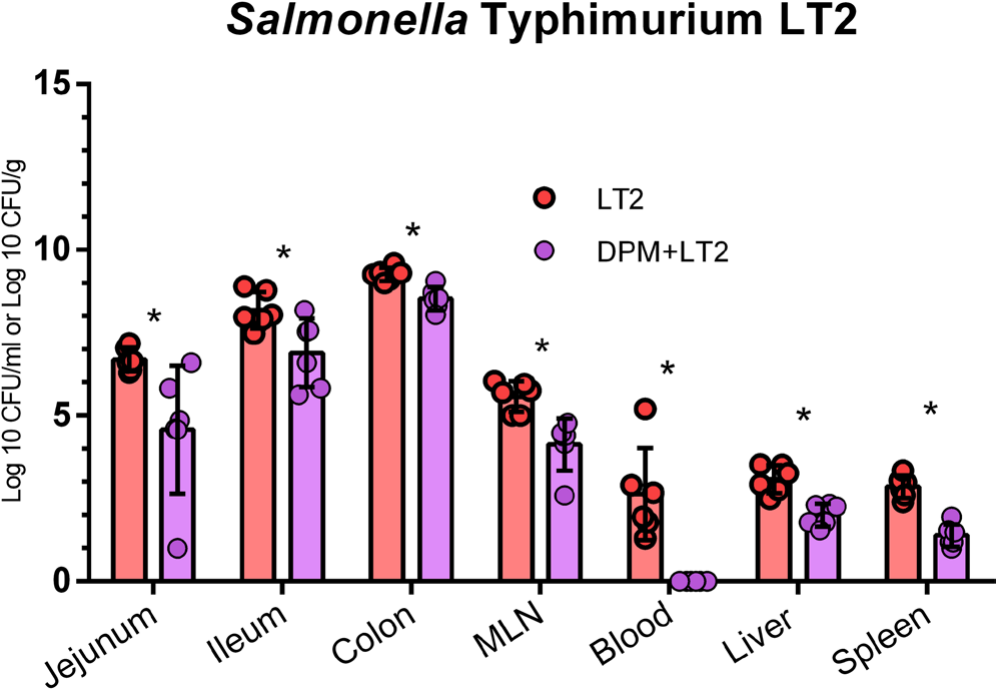
*S*. Typhimurium LT2 log CFU in organs. Comparison of LT2 log CFU/mL or log CFU/g in the jejunum, ileum, colon, mesenteric lymph nodes (MLN), blood, liver, and spleen between the piglets infected with *S*. Typhimurium LT2 (LT2; red dots) and piglets pre‐colonized with DPM and subsequently infected with *S*. Typhimurium LT2 (DPM + LT2; violet dots). Six samples per group were analyzed, and statistical significance was determined using a Student's t‐test. The values are presented as individual dots and mean ± SEM, and significant differences (*p <* 0.05) are denoted by an asterisk (*).

LT2 colonized the intestine at increasing log CFU/mL densities in lavaged intestinal digesta in the jejunum, ileum, and colon, respectively (Figure 3). *Salmonella* translocated to the mesenteric lymph nodes, blood, liver, and spleen. Pre‐colonization with DPM significantly decreased the colonization and translocation of LT2 CFU in the following *Salmonella* infection in the DPM + LT2 piglets in comparison with the piglets infected with LT2 without the pre‐colonization (LT2). This decrease was significant in all observed organs, and LT2 was not detected in the blood of the DPM + LT2 group 24 h post‐infection.

### 3.3 Macroscopic Differences in the Gastrointestinal Tract Between GF and LT2 Piglets

**FIGURE 4:**
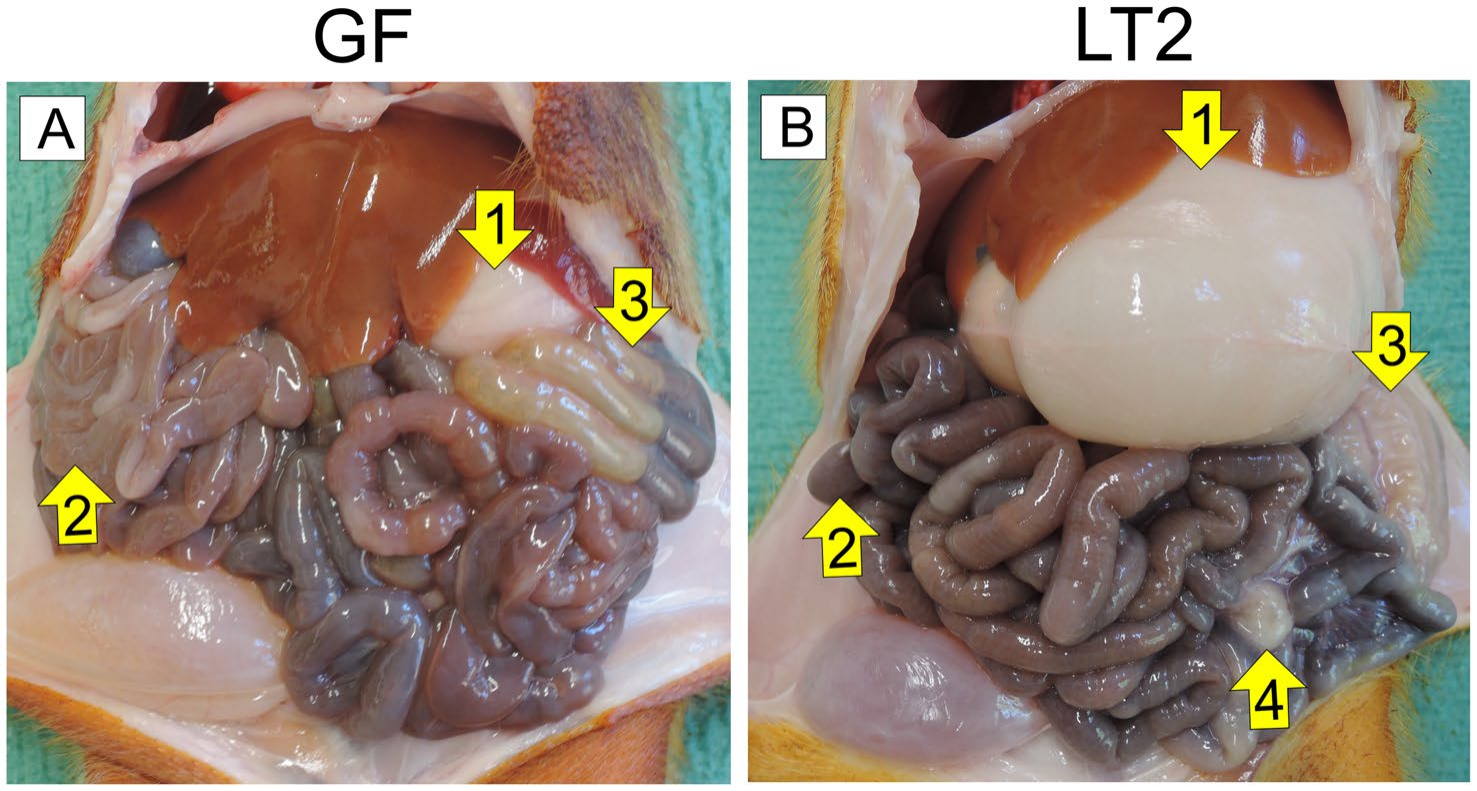
Gastrointestinal tracts of GF and LT2‐infected GN piglets. GF piglets (A) show 6 h after the last feeding, empty stomach (1) and jejunum (2), but colon filled with the digesta (3). LT2 piglets (B) show the stomach full of precipitated milk diet (1), jejunum filled with digesta (2), empty colon (3), and highly enlarged mesenteric lymph nodes (4).

After 6 h of starvation, the GF piglet showed an empty stomach and proximal jejunum at autopsy. The distal jejunum, ileum, and colon were filled with digesta (Figure 4A). Twenty‐four hours after the infection with LT2, the LT2‐infected piglets had a full stomach of precipitated milk diet; the jejunum and, to a lower degree, also the ileum contained digesta, but the colon was empty (Figure 4B). The mesenteric lymph nodes of the infected piglets were highly enlarged compared to their GF counterparts (Figure 4B).

### 3.4 Acidic Mucin‐Producing Goblet Cells

**FIGURE 5:**
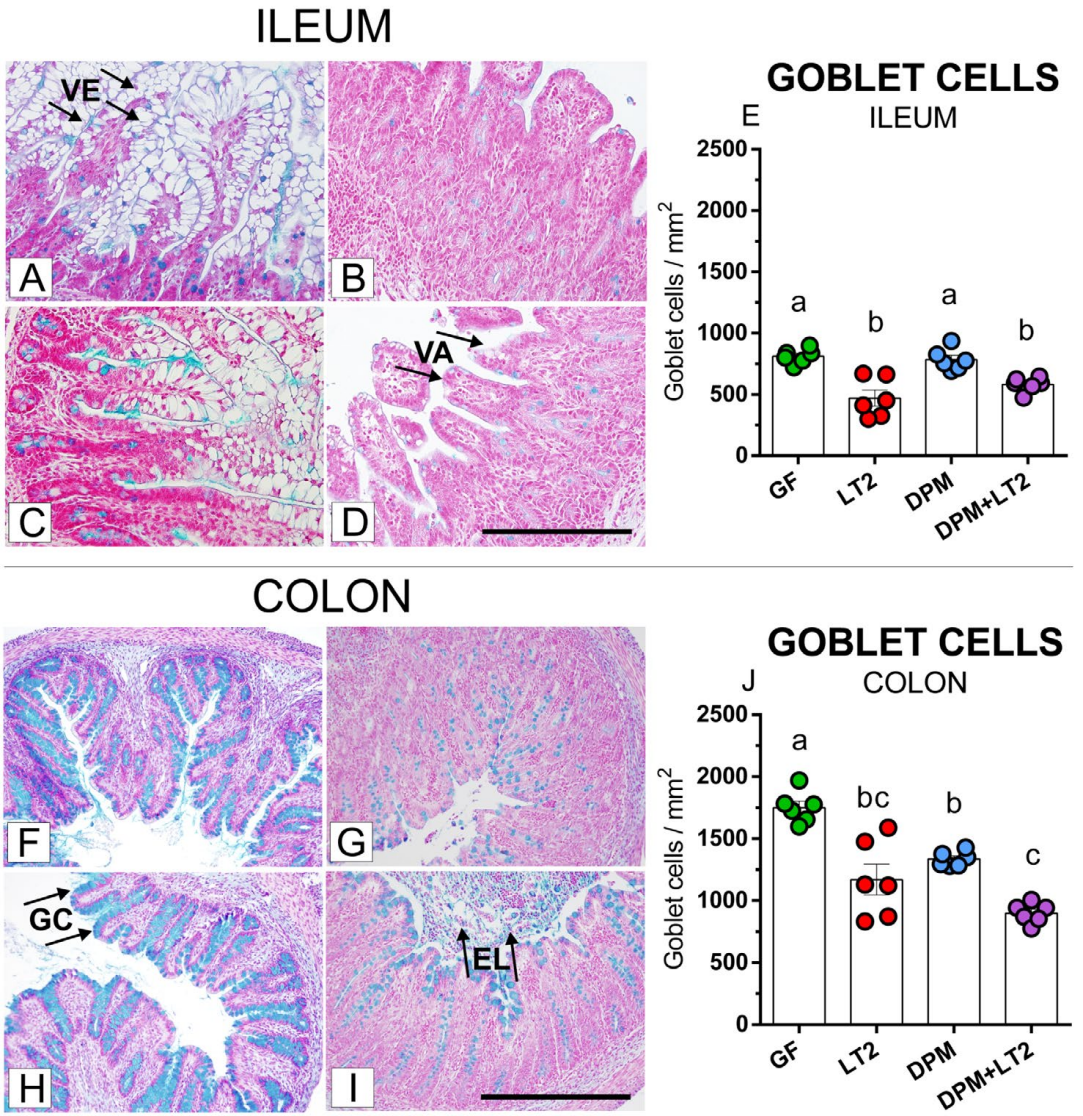
Acidic mucin‐producing goblet cells in the ileum and colon. (i) germ‐free (GF; A,F), (ii) infected with *S*. Typhimurium LT2 for 24 h (LT2; B,G), (iii) colonized with defined pig microbiota (DPM; C,H), (iv) colonized with DPM and infected with LT2 for 24 h (DPM + LT2; D,I). Number of acid mucin‐producing goblet cells per mm^2^ in the ileum (E) and colon (J) of one‐week‐old GN piglets. The scale bars (D, I) depict 100 μm. The values are presented as individual dots and mean ± SEM. Statistical analysis was performed using one‐way ANOVA followed by Tukey's post hoc multiple comparison test. Groups with *p* < 0.05 are indicated by different letters above the columns. Each group consisted of six samples. VE—vacuolated enterocytes, VA—villus atrophy, GC—goblet cells, and EL—exudate in lumen containing neutrophils.

The staining of the ileum (Figure 5 A‐D) and colon (Figure 5 F‐I) for the presence of acidic mucin showed intestinal structure and acidic mucin‐producing goblet cells. The ileum of the GF piglets (A) showed rich vacuolated enterocytes. Comparable goblet cell densities (E) were found in piglets colonized with DPM, but vacuolated enterocytes partially disappeared (C). The vacuolated enterocytes disappeared in both groups of LT2‐infected piglets (LT2; B and DPM + LT2; D), and the ileum exhibited inflammatory signs, primarily characterized by villi atrophy and edema, and significantly decreased goblet cell densities (E).

In the colon (Figure 5 F‐I), the presence of bacteria (LT2, DPM, and DPM + LT2 groups) significantly decreased goblet cell density compared to the GF piglets (J). The *Salmonella* infection (LT2; G and DPM + LT2; I) induced inflammation in the colon, for example, exudate in the lumen containing neutrophils. In contrast, no inflammation was observed in the colon of the piglets colonized with DPM (H).

### 3.5 Villin Expression in the Colon

**FIGURE 6:**
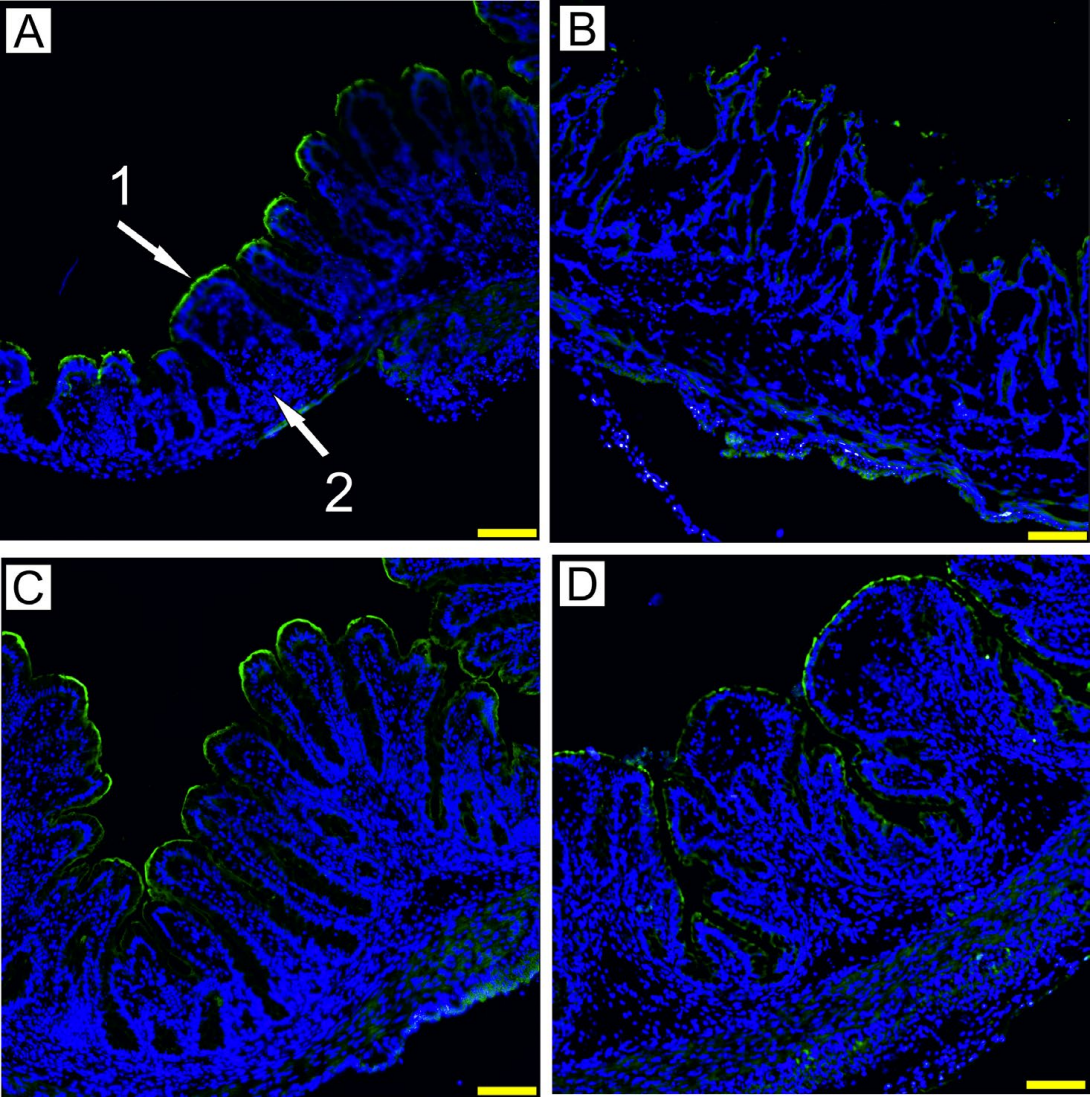
Expression of villin in the colon of GN piglets. (i) germ‐free (GF; A), (ii) piglets infected with S. Typhimurium LT2 (LT2; B), (iii) piglets colonized with DPM (DPM; C), and (vi) DPM piglets infected with *S*. Typhimurium (DPM + LT2; D). Green fluorescence (arrow 1) depicts the presence of villin, and blue fluorescence (arrow 2) depicts cell nuclear DNA. The scale bar is 100 μm.

A sharp villin border was found in the colon of the GF, DPM, and DPM + LT2 piglet groups (Figure 6A,C,D). In contrast, this border was disrupted by *Salmonella* infection in the LT2 piglets (Figure 6B).

### 3.6 Villin, Claudin‐1, and Occludin mRNA Expression in the Intestine

**FIGURE 7:**
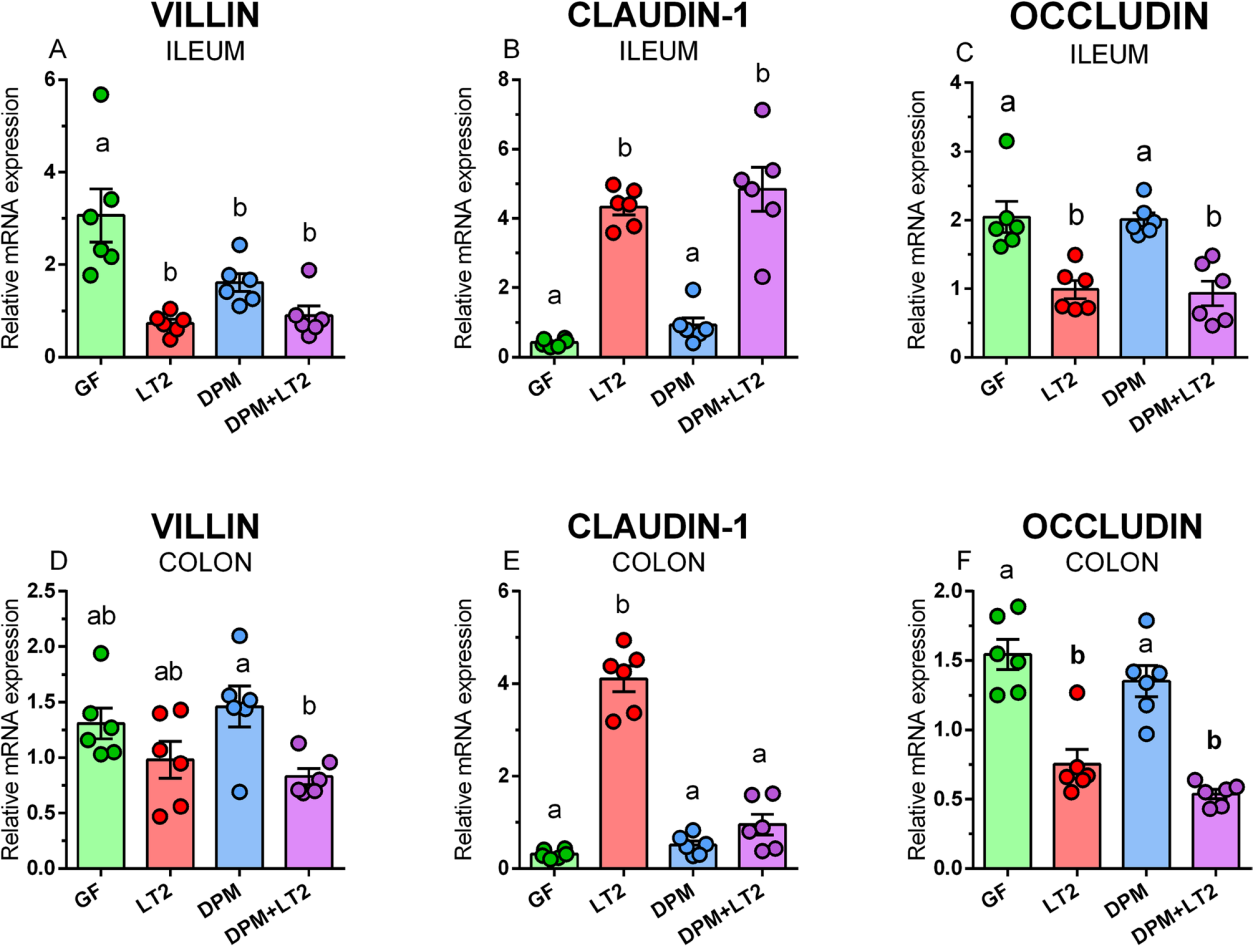
Expression of villin, claudin‐1, and occludin mRNA in the intestine of the GN piglets. Villin (A,D), claudin‐1 (B,E), and occludin (C,F) in the ileum (A‐C) and colon (D‐F) of GN piglets: (i) germ‐free (GF), (ii) infected with *S*. Typhimurium LT2 (LT2), (iii) colonized with DPM (DPM), and (iv) colonized with DPM and infected with LT2 (DPM + LT2). The values are presented as individual dots and mean ± SEM. Statistical analysis was performed using one‐way ANOVA followed by Tukey's post hoc multiple comparison test. Groups with *p* < 0.05 are indicated by different letters above the columns. Each group consisted of six samples.

Villin mRNA expression was significantly decreased in all bacteria‐colonized piglet groups in the ileum and in DPM + LT2 piglets in the colon (Figure 7A,D). Claudin‐1 mRNA expression was significantly increased in *Salmonella*‐infected piglets (LT2 and DPM + LT2) in the ileum and in the LT2 group in the colon (Figure 7B,E). Occludin mRNA expression was significantly downregulated in both *Salmonella*‐infected groups in the ileum and colon (Figure 7C,F).

### 3.7 LPS Levels in the Ileum, Colon, and Plasma

**FIGURE 8:**
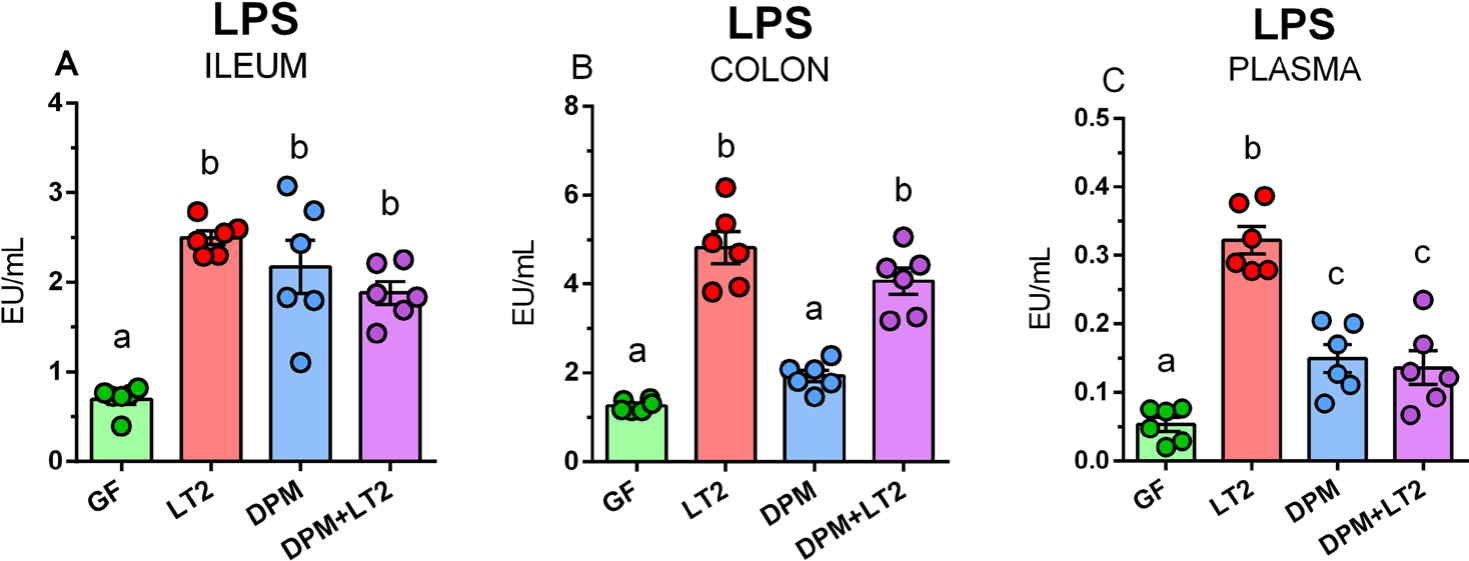
LPS in the intestine and plasma. LPS levels in the ileum (A), colon (B), and plasma (C) of (i) germ‐free (GF) piglets, (ii) piglets infected with *S*. Typhimurium LT2 (LT2), (iii) piglets colonized with DPM (DPM), and (iv) DPM piglets infected with *S*. Typhimurium LT2 (DPM + LT2). The values are presented as individual dots and mean ± SEM. Statistical analysis was performed using one‐way ANOVA followed by Tukey's post hoc multiple comparison test. Groups with *p* < 0.05 are indicated by different letters above the columns. Each group consisted of six samples.

A milk diet contains a negligible amount of LPS. The LPS levels in the ileum, colon, and plasma of the bacteria‐colonized piglets were higher than in the GF piglets (Figure 8). These differences, with the exception of the colon in the DPM group, were significant.

### 3.8 TLR4, MD‐2, LBP, CD14, MyD88, and TRIF mRNA Expression in the Ileum and Colon

**FIGURE 9:**
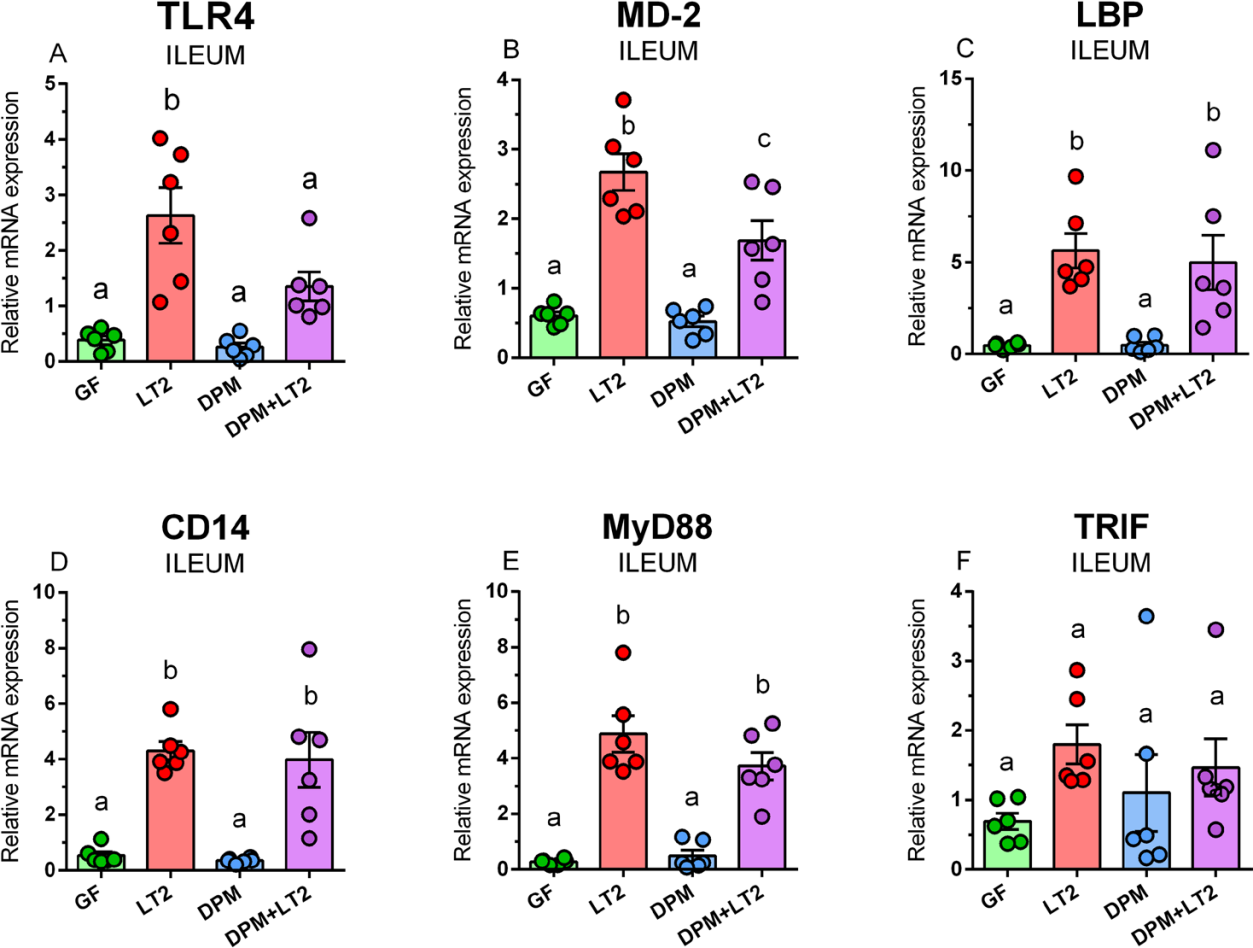
TLR4/MD‐2 signaling in the ileum. TLR4 (A), MD2 (B), LBP (C), CD14 (D), MyD88 (E), and TRIF (F) mRNA expression (fold change) in the ileum of (i) germ‐free (GF) piglets, (ii) piglets infected with *S*. Typhimurium LT2 (LT2), (iii) piglets colonized with DPM (DPM), and (iv) DPM piglets infected with *S*. Typhimurium LT2 (DPM + LT2). The values are presented as individual dots and mean ± SEM. Statistical analysis was performed using one‐way ANOVA followed by Tukey's post hoc multiple comparison test. Groups with *p* < 0.05 are indicated by different letters above the columns. Each group consisted of six samples.

The infection with *S*. Typhimurium LT2 significantly increased TLR4 mRNA in the ileum compared to the other groups (Figure 9A). The colonization with DPM did not influence TLR4 mRNA expression compared to the GF control. The previous colonization with DPM significantly limited TLR4 mRNA expression after subsequent infection with LT2 (DPM + LT2) in the piglets infected with LT2 without the previous DPM treatment (LT2). The infection with LT2 significantly upregulated MD‐2 compared to other piglet groups (Figure 9B). The previous colonization with DPM limited the upregulation in the DPM + LT2 group, but the differences between non‐infected piglets (GF and DPM) and the piglets infected with LT2 only were significant. LBP (Figure 9C), CD14 (Figure 9D), and MyD88 (Figure 9E) mRNA expression showed similar profiles. Both infected piglets (LT2 and DPM + LT2) showed significant upregulation compared to non‐infected groups (GF and DPM). TRIF mRNA expression in all groups was comparable (Figure 9F).

**FIGURE 10:**
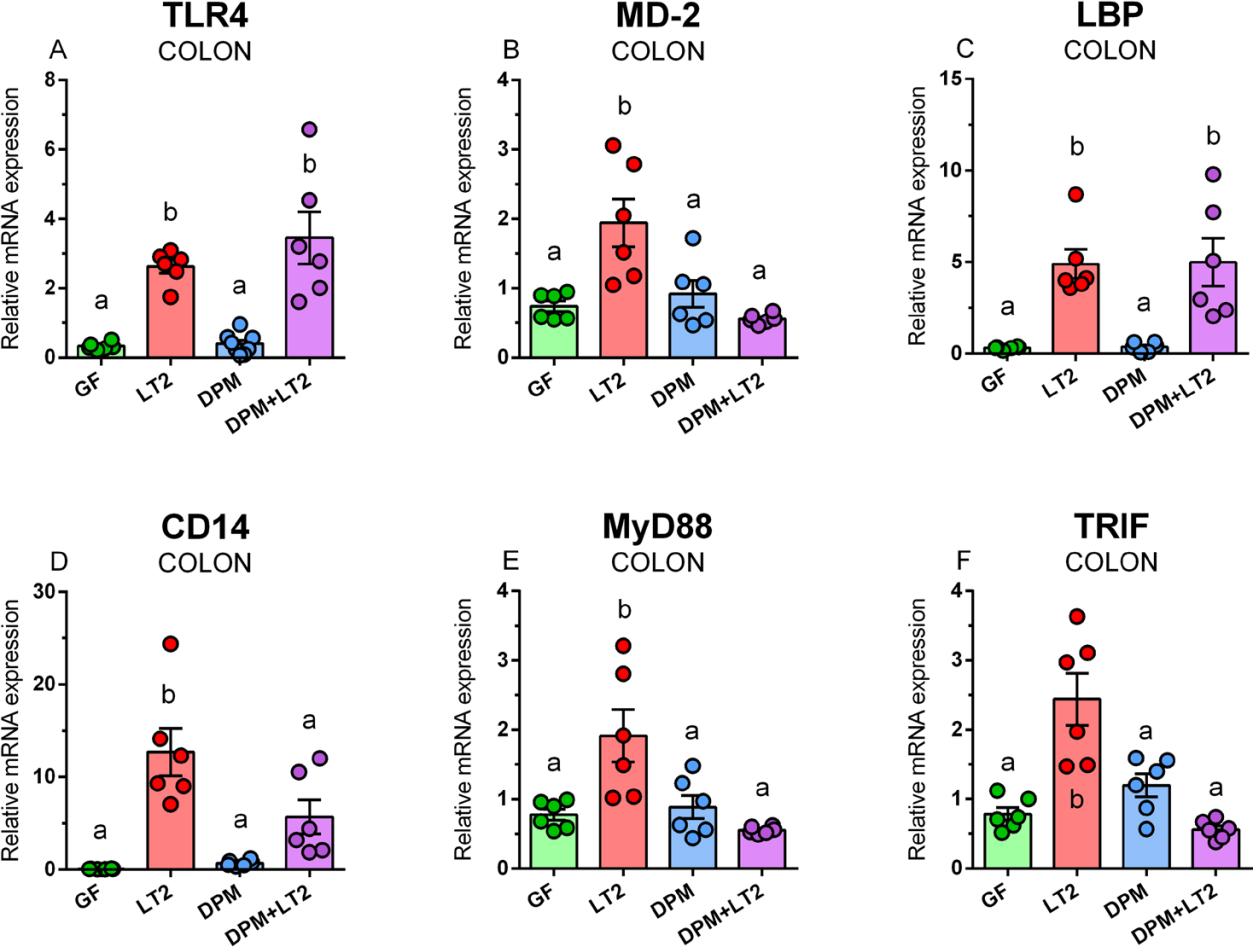
TLR4/MD‐2 signaling in the colon. TLR4 (A), MD2 (B), LBP (C), CD14 (D), MyD88 (E), and TRIF (F) mRNA expression (fold change) in the colon of (i) germ‐free (GF) piglets, (ii) piglets infected with *S*. Typhimurium LT2 (LT2), (iii) piglets colonized with DPM (DPM), and (iv) DPM piglets infected with *S*. Typhimurium LT2 (DPM + LT2). The values are presented as individual dots and mean ± SEM. Statistical analysis was performed using one‐way ANOVA followed by Tukey's post hoc multiple comparison test. Groups with *p* < 0.05 are indicated by different letters above the columns. Each group consisted of six samples.

In the colon, TLR4 mRNA was significantly upregulated in both LT2‐infected groups (Figure 10A). A similar trend, without the ability of the previous colonization with DPM to prevent upregulation of mRNA expression, showed LBP mRNA expression (Figure 10C). In contrast, infection with LT2 upregulated MD2 (Figure 10B), CD14 (Figure 10D), MyD88 (Figure 10E), and TRIF mRNA expression (Figure 10F). However, previous colonization with DPM prevented the upregulation of these mRNA expressions.

### 3.9 Soluble TLR4, TLR2, And CD14 in the Ileum and Colon

**FIGURE 11:**
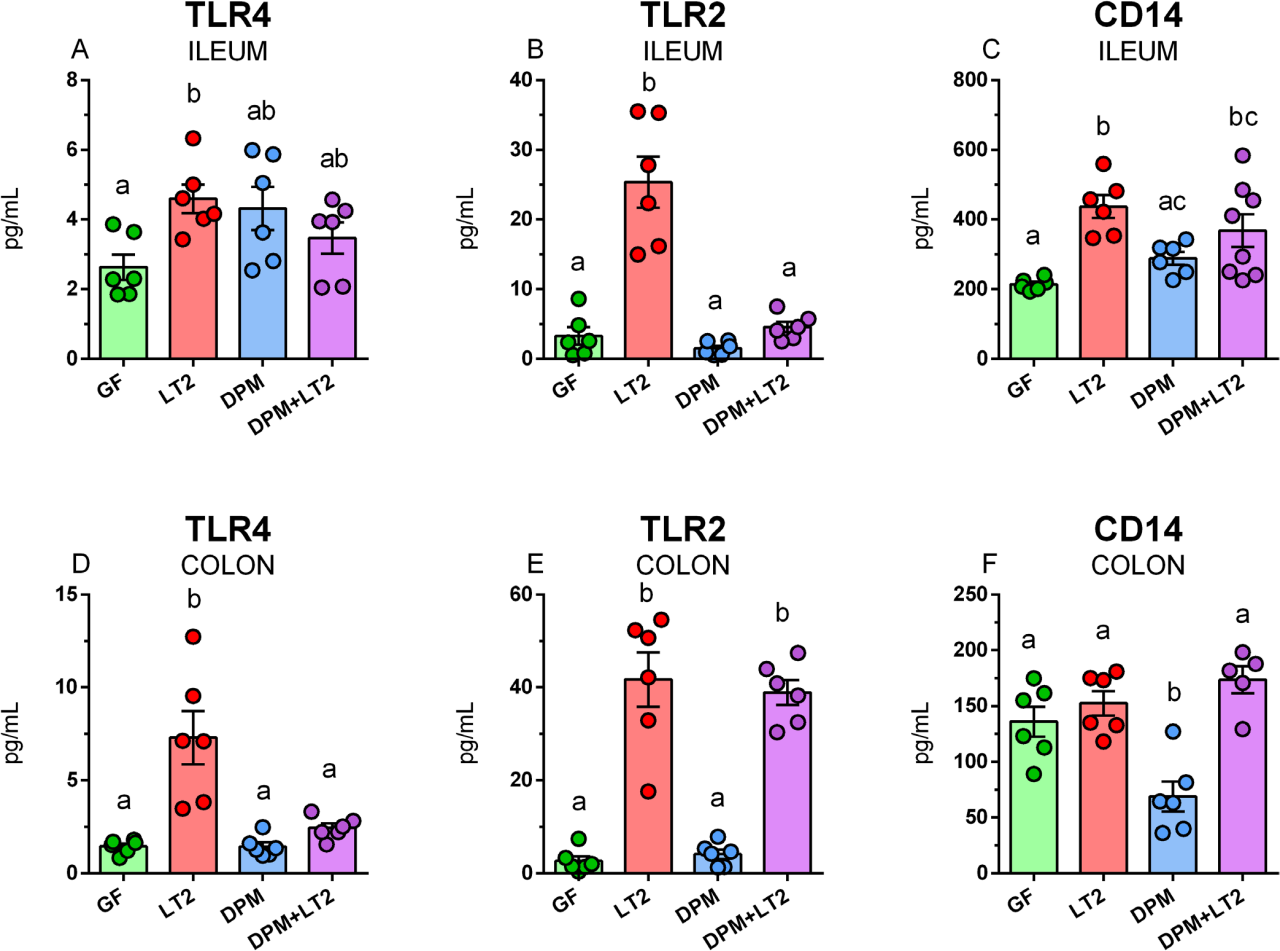
Soluble TLR4, TLR2, and CD14 in the intestine. Soluble TLR4 (A and D), TLR2 (B and E), and CD14 (C and F) levels in the ileum (A‐C) and colon (D‐F) of (i) germ‐free (GF) piglets, (ii) piglets infected with *S*. Typhimurium LT2 (LT2), (iii) piglets colonized with DPM (DPM), and (iv) DPM piglets infected with *S*. Typhimurium LT2 (DPM + LT2). The values are presented as individual dots and mean ± SEM. Statistical analysis was performed using one‐way ANOVA followed by Tukey's post hoc multiple comparison test. Groups with *p* < 0.05 are indicated by different letters above the columns. Each group consisted of six samples.

The presence of bacteria in the ileum stimulates the release of TLR4 into digesta (Figure 11A). This release was significant only in the case of the LT2 group compared to GF piglets. TLR2 presence in the ileum increased only in the LT2 group, but other piglet groups were comparable with GF piglets (Figure 11B). Soluble CD14 levels in the intestine were significantly increased in both groups infected with LT2 (LT2 and DPM + LT2; (Figure 11C). In the colon, TLR4 was significantly increased in the LT2 piglets only (Figure 11D), but TLR2 in both LT2‐infected groups (Figure 11E). GF, LT2, and DPM + LT2 groups had comparable levels of CD14, but they were significantly lower in the DPM group compared to all other groups Figure 11F).

**FIGURE 12:**
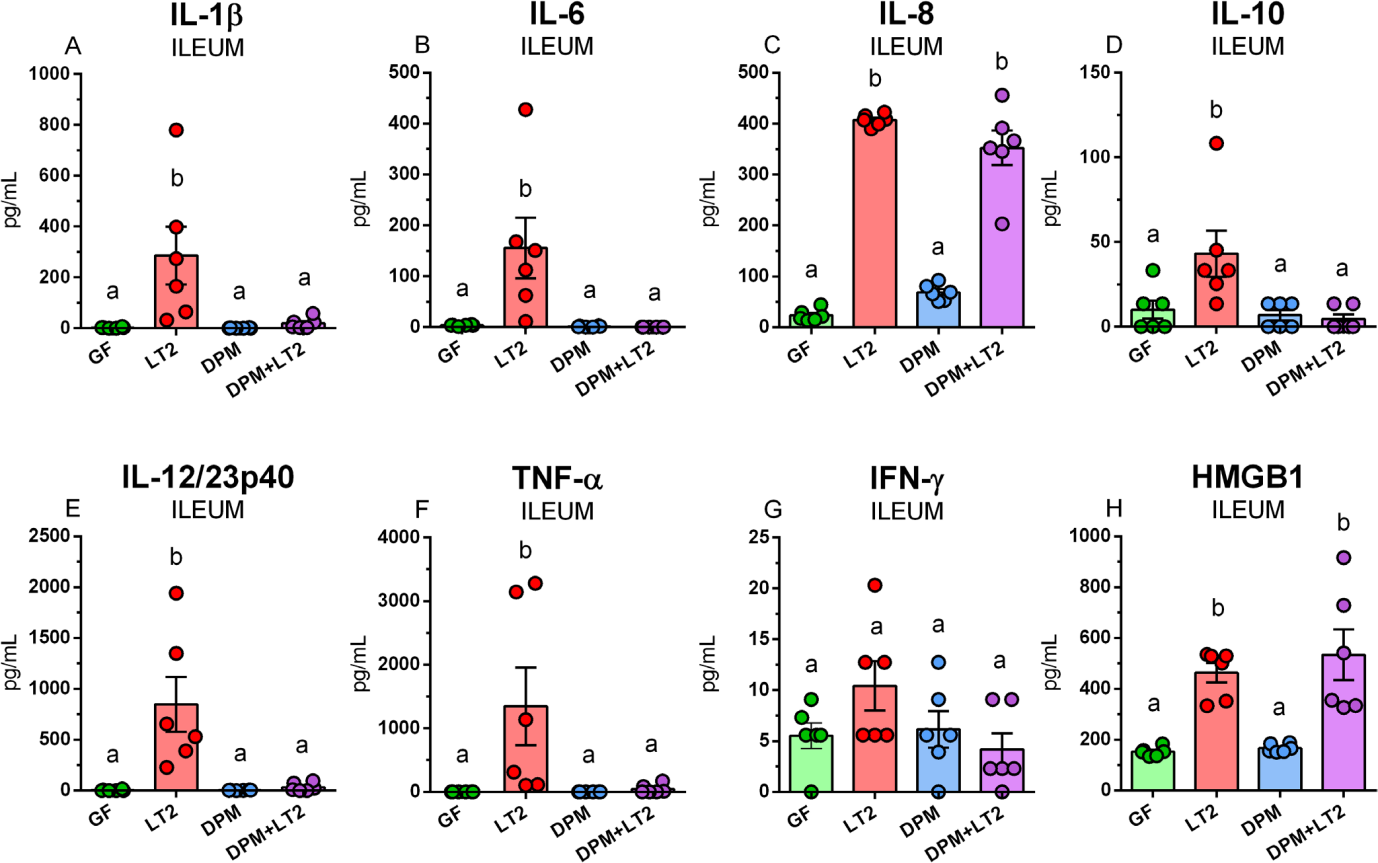
IL‐1*β*, IL‐6, IL‐8, IL‐10, IL‐12/23p40, TNF‐*α*, IFN‐*γ*, and HMGB1 in the ileum. IL‐1*β* (A), IL‐6 (B), IL‐8 (C), IL‐10 (D), IL‐12/23p40 (E), TNF‐*α* (F), IFN‐*γ* (F), and HMGB1 (H) in the ileum of (i) germ‐free (GF) piglets, (ii) piglets infected with *S*. Typhimurium LT2 (LT2), (iii) piglets colonized with DPM (DPM), and (iv) DPM piglets infected with *S*. Typhimurium LT2 (DPM + LT2). The values are presented as individual dots and mean ± SEM. Statistical analysis was performed using one‐way ANOVA followed by Tukey's post hoc multiple comparison test. Groups with *p* < 0.05 are indicated by different letters above the columns. Each group consisted of six samples.

DPM did not upregulate the levels of observed inflammatory markers compared to GF piglets (Figure 12). Levels of inflammatory cytokines IL‐1*β* (Figure 12A), IL‐6 (Figure 12B), IL‐8 (Figure 12C), IL‐10 (Figure 12D), IL‐12/23p40 (Figure 12E), TNF‐*α* (Figure 12F), and HMGB1 (Figure 12H) were significantly upregulated by infection with *S*. Typhimurium in the LT2 group. Similar upregulation was observed also in IL‐8 (Figure 12C) and HMGB1 (Figure 12H) in the DPM + LT2 piglets.

**FIGURE 13:**
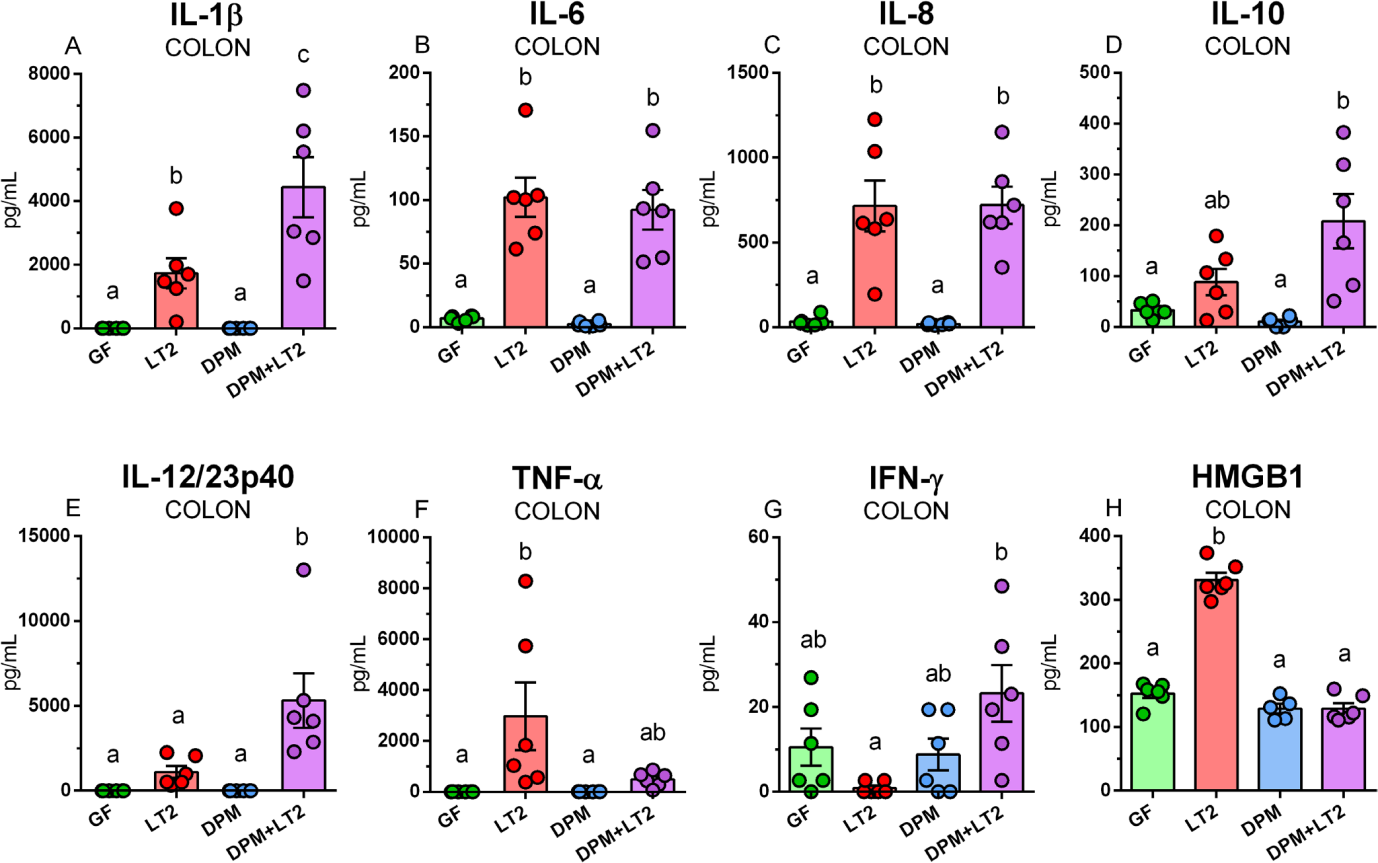
IL‐1*β*, IL‐6, IL‐8, IL‐10, IL‐12/23p40, TNF‐*α*, IFN‐*γ*, and HMGB1 in the colon. IL‐1*β* (A), IL‐6 (B), IL‐8 (C), IL‐10 (D), IL‐12/23p40 (E), TNF‐*α* (F), IFN‐*γ* (F), and HMGB1 (H) in the colon of (i) germ‐free (GF) piglets, (ii) piglets infected with *S*. Typhimurium LT2 (LT2), (iii) piglets colonized with DPM (DPM), and (iv) DPM piglets infected with *S*. Typhimurium LT2 (DPM + LT2). The values are presented as individual dots and mean ± SEM. Statistical analysis was performed using one‐way ANOVA followed by Tukey's post hoc multiple comparison test. Groups with *p* < 0.05 are indicated by different letters above the columns. Each group consisted of six samples.

Levels of inflammatory mediators in the colon in the GF and DPM piglet groups were comparable in all observed mediators (Figure 13). The LT2 group had significantly higher levels of IL‐1*β* (Figure 13A), IL‐6 (Figure 13B), IL‐8 (Figure 13C), TNF‐*α* (Figure 13F), and HMGB1 (Figure 13H), and the DPM + LT2 group in IL‐1*β* (Figure 13A), IL‐6 (Figure 13B), IL‐8 (Figure 13C), IL‐10 (Figure 13D), IL‐12/23p40 (Figure 13E), and IFN‐*γ* (Figure 13G).

## 4 Discussion

Placental transfer of maternal immunoglobulins, their levels, and their isotype composition in newborns at birth depend on the type of placenta. The human hemochorial placenta allows the prenatal transfer of protective class G immunoglobulins (IgG). In contrast, placentas without reduced tissue layers between the trophoblast and the mother's blood do not allow it [30, 31]. Thus, newborn piglets must postnatally intake enough colostrum containing immunoglobulins and immunocytes as soon as possible after birth to survive/thrive in pigpen conditions [32]. Surgically derived (germ‐free; GF) piglets [33] can survive and thrive in sterile conditions without the intake of colostrum [25]. The breeding of piglets in gnotobiotic (GN; microbiologically controlled) conditions allows modulating immunocompetence, for example, by the addition of bovine colostrum or porcine serum containing immunoglobulins [34] and the seeding of GF animals by a defined (synthetic) microbiota [11].

The colonization of the piglets with DPM did not negatively affect piglet health. This pre‐colonization with DPM significantly altered *Salmonella* multiplication in the intestine and *Salmonella* translocation into the organs. These piglets exhibited alleviation of the profuse diarrhea characteristic of the *Salmonella*‐infected LT2 group piglets. Autopsy findings showed cessation of intestinal peristalsis, a sign that can occur as part of the clinical manifestations of salmonellosis in humans [35], but pre‐colonization with DPM partially prevented it.

We observed the persistence of fetal‐type vacuolated enterocytes in the ileum of one‐week‐old GF and DPM piglets, which are typical for newborn piglets [36]. These types of enterocytes persisted in GN piglets of the same age colonized with commensal lactobacilli, but disappeared in the piglets colonized with probiotic *Escherichia coli* Nissle 1917 [37]. Similarly, vacuolated enterocytes persisted in two‐week‐old GF piglets in an experiment by others, but they disappeared in their counterparts that were conventionalized with adult pig feces [38].

The intestinal epithelium is a single layer of epithelial cells, including various functionally specialized enterocytes that differ in their composition across the intestine [39]. Adjacent enterocytes are connected at their apical regions by tight junction (TJ) proteins, such as claudins and occludin [40], and are covered with mucins produced by goblet cells. The mucin layer serves as the first line of defense against bacterial translocation [41]. Enteric pathogens, such as *Salmonella enterica* , can disrupt the intestinal barrier [13]. The presence of bacteria in the intestine had a stimulatory effect on mucin production in originally GF mice that were later colonized with undefined microbiota [42]. Mucin 2 (MUC2) is the most abundant mucin in the intestine [41, 43]. However, no differences in MUC2 mRNA expression among GF, *Salmonella*‐infected, and *Bifidobacterium boum* ‐colonized GN piglets were found [28]. Thus, in this work, we focused solely on the staining density of acidic mucin‐producing cells. Mucins are continuously released at a baseline level; however, goblet cells can store mucin granules and release them into the lumen to protect the epithelial barrier from direct contact with threats, such as bacteria [39]. The presence of *S*. Typhimurium (LT2 and DPM + LT2) decreases the goblet cell density in the ileum. Exocytosis of mucin can be a reason for the lower density of acidic mucin‐producing goblet cells [39], as we found. In the colon, all bacteria‐colonized GN piglet groups showed a decrease in goblet cell density compared to the GF group.

Cytoskeletal protein villin is involved in the balance between actin polymerization and actin severing and facilitates the initial steps of *Salmonella* invasion [44]. Villin can be used as a marker of enterocyte differentiation and participates in the restitution of damaged epithelia [45]. The presence of bacteria downregulated villin mRNA expression. At the protein level, the GF and DPM piglet groups exhibited a sharp villin border in the apical part of colonocytes, but this border was disrupted by *S*. Typhimurium infection in the LT2 group. The pre‐colonization with DPM (DPM + LT2) ameliorated *Salmonella*‐caused villin disruption, and villin protein expression was almost as clearly expressed as in the GF and DPM groups. This finding confirmed the protective role of DPM.

*S*. Typhimurium causes diarrhea in humans [46] and pigs [14], which alters the transport of electrolytes [47]. Claudin‐1 belongs to the “sealing” claudins that manage electrolytes, and their loss is related to diarrhea [40]. Claudin‐1 mRNA expression in the ileum was upregulated in both *S*. Typhimurium‐infected piglet groups, but pre‐colonization with DPM prevented this upregulation. We hypothesize that the upregulation of claudin‐1 mRNA expression in the ileum is an attempt to seal the disrupted intestinal barrier. The composition of the microbiota in the ileum and colon of conventional piglets differs [48], and the presence of various bacterial strains and total CFU counts in these parts of the intestine also differed in GF piglets colonized with DPM [23]. Thus, it is possible to expect different effectiveness in suppressing the detrimental effect of *S*. Typhimurium in the ileum and colon [12]. Occludin is related to the transmission of macromolecules. Its disappearance facilitates the leakage of large molecules from the intestine [49]. In contrast to claudin‐1, occludin mRNA expression was downregulated by infection, and pre‐colonization with DPM did not prevent it. Experiments with pre‐monocolonization of GF piglets with various bacteria [27, 28, 50] showed that only the widely used probiotic *Escherichia coli* Nissle 1917 was highly protective against infection with *S*. Typhimurium [27]. DPM showed comparable protective effectiveness with *E. coli* Nissle 1917.

LPS is a component of the cell wall of Gram‐negative bacteria that is released from dead bacteria [51]. We measured LPS concentrations in the intestines and plasma of all piglets. The “basal” LPS levels in the piglets can be attributed to the presence of a low amount of LPS in the milk diet that we detected. It is difficult to destroy LPS, and its varying amounts were found in infant milk formulas [52]. We measured increased LPS levels in the intestinal content of piglets infected with *Salmonella*. Currently, we are unable to explain the surprisingly increasing LPS levels in the DPM piglets in the ileum. The simplest explanation could be contamination with Gram‐negative bacteria. However, a significant increase of LPS in the DPM compared to GF piglets did not occur in the colon to confirm this possibility. A speculative explanation is the influence of bile acids or other biological compounds metabolized by the microbiota and their differential occurrence and abundance in different parts of the intestine [1]. In plasma, the LT2 group LPS levels were high compared to those of other piglet groups. The low amount of LPS in plasma in the DPM + LT2 piglets can be explained by the pre‐colonization with DPM, which prevented bacteremia in these piglets.

**FIGURE 14:**
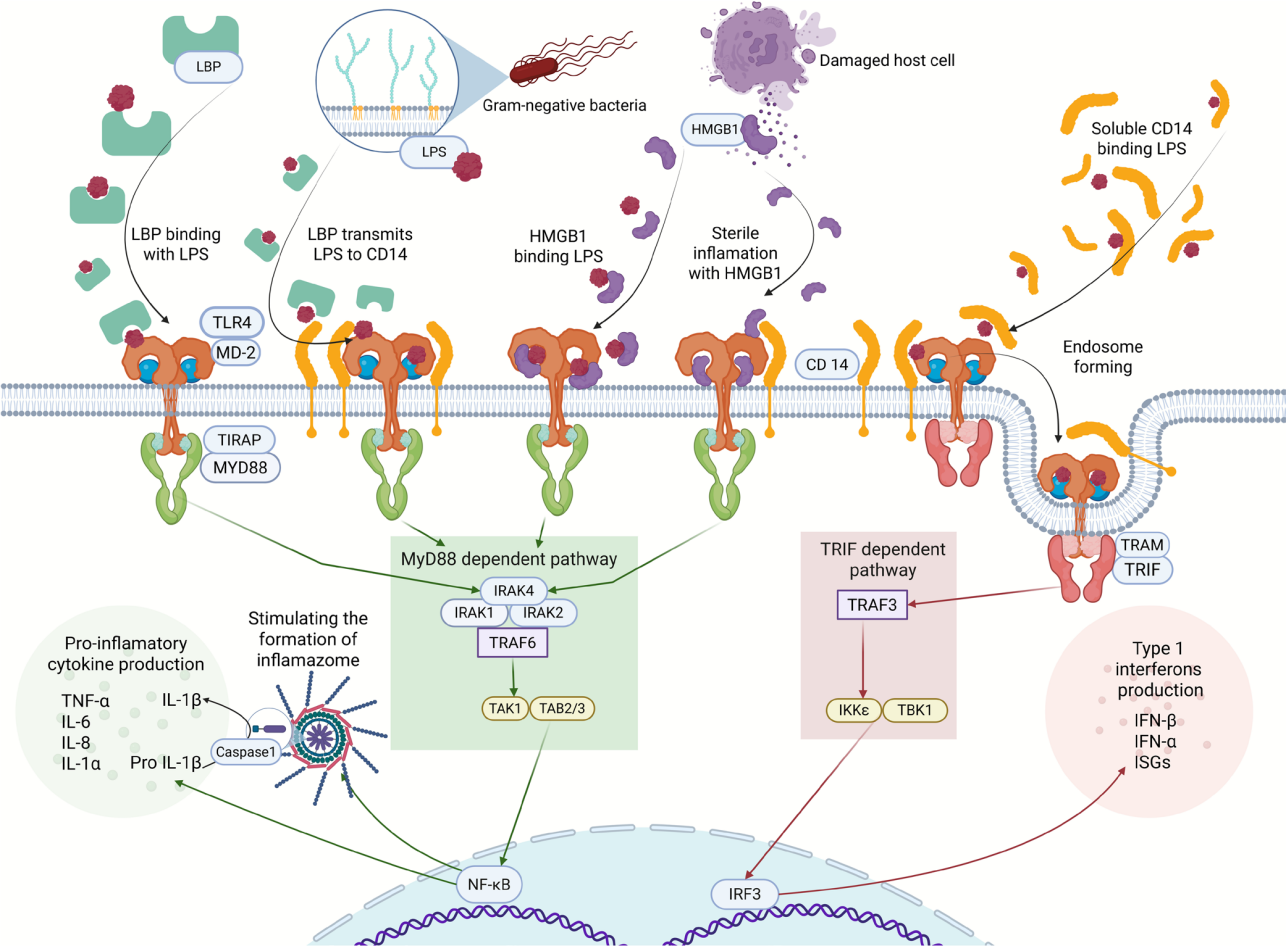
TLR4‐MD2 complex signaling pathway. LPS (lipopolysaccharide) is trapped and concentrated by LBP (LPS‐binding protein), which is directly bound to the TLR4/MD‐2 complex or CD14 (TLR coreceptor) and moves to the TLR4/MD‐2 complex. Membrane‐bound TLR4 signals via MyD88, whereas endosomal TLR4 signals via the TRIF adaptor protein. Inflammatory cytokines and type 1 interferons are produced after TLR4/MD‐2 complex‐mediated LPS recognition. TLR4 is a multiligand receptor, and another of its ligands is HMGB1 (high mobility group box 1), which can bind LPS similarly to LBP. The figure was created with BioRender.com (RRID:SCR_018361).

Toll‐like receptor 4 (TLR4) is a multiligand receptor of PAMP and DAMP ligands [17, 18]. Thus, a modulation of the TLR4 signaling pathway has therapeutic potential for many diseases [53]. In infectious immunology, TLR4 is associated primarily with the recognition of lipopolysaccharide (LPS) [18], but sterile inflammation can also be caused by HMGB1, which is another TLR4 ligand [17] (Figure 14).

LBP binds and concentrates LPS, and alone or in conjunction with CD14, transports it to the TLR4/MD‐2 complex [18, 51]. The infection with *S*. Typhimurium significantly increased TLR4, MD‐2, LBP, CD14, and MyD88 mRNA in both parts of the intestine. The pre‐colonization with DPM suppressed this upregulation of MD‐2, CD14, and MyD88 mRNA expression in the colon. The results revealed the activation of the TLR4/MD‐2 complex and downstream signaling by the infection [18]. We again related it to the different colonization [23], as well as differences in the immune system in these parts of the intestine, for example, the dominance of Peyer's patches in the ileum in contrast to isolated lymphoid follicles in the colon [54, 55].

Activation of the TLR4 pathway results in the production of inflammatory cytokines or type I interferons, depending on the localization of TLR4/MD‐2 on the cell membrane or in the endosome and the use of MyD88‐dependent or TRIF‐dependent adaptor molecules, respectively [18]. Inflammatory cytokines provide regulatory signals to control the immune response. Systemic cytokine levels may not accurately reflect local cytokine levels because of their short half‐lives [56]. Thus, we focused on cytokines in the intestinal lumen. Pro‐inflammatory IL‐1*β*, IL‐6, IL‐8, IL‐12/23p40, TNF‐*α*, and IFN‐*γ*, and anti‐inflammatory (regulatory) cytokine IL‐10 play key roles in the acute phase of sepsis [57]. HMGB1 is believed to be a critical molecule in the pathogenesis of Gram‐negative sepsis [58].

Disruption of the TLR4 signaling pathway can downregulate pro‐inflammatory signaling and excessive production of inflammatory mediators. Neutralization with anti‐CD14 antibodies ameliorated the induction of inflammatory mediators in a pig model of *Escherichia coli* caused Gram‐negative sepsis [59]. We detected negligible levels of inflammatory cytokines in the ileum of GF control piglets. Commensal microbiota plays a crucial role in the normal development of the immune system; however, it can also trigger inflammatory responses in immunodeficient hosts [60]. The colonization with DPM did not upregulate the constitutive levels of the inflammatory cytokines and HMGB1 that were found in the non‐stimulated GF piglet. It is in accordance with the histological findings and the absence of enterocolitis signs, except for mild and temporary diarrhea. In contrast, the infection with *Salmonella* significantly upregulated levels of all observed inflammatory cytokines and HMGB1, except for IFN‐*γ*. The pre‐colonization with DPM suppressed the upregulation of most of the analyzed cytokines after infection with *Salmonella*, and maintained them at comparable levels to those of the GF piglets. In contrast, the levels of IL‐8 (CXCL‐8) and HMGB1 were upregulated. IL‐8 is a chemokine (chemotactic cytokine) that attracts neutrophils to the inflammatory site and activates them [61]. Gnotobiotic piglets that were pre‐colonized with avirulent *Salmonella* Infantis or *S*. Typhimurium strains were resistant to the subsequent infection with virulent *S*. Typhimurium strains [62, 63]. The beneficial effect of avirulent *Salmonella* serovars was attributed to their ability to induce local levels of IL‐8. In our current study, DPM did not induce local IL‐8 levels. It is possible that comparable levels of IL‐8 due to increased colonization resistance could reflect a beneficial effect of DPM on the piglets [12]. Conversely, detrimental effects are likely mediated by other inflammatory cytokines that were upregulated in the LT2 piglets but not in DPM + LT2 piglets. Intestinal HMGB1 levels were related to the severity of enteric infections in GN piglets [64], and its increased fecal levels were detected in newborn infants with necrotizing enterocolitis [65]. Therefore, we hypothesize that increased levels of HMGB1 may predict the development of intestinal inflammation in the experiment, which would last longer than 24 h post‐infection. The levels of most cytokines, but not HMGB1, indicated less effectiveness of the pre‐colonization with DPM in the colon than in the ileum.

TLRs consist of an extracellular domain responsible for detecting PAMPs and DAMPs, a transmembrane region, and an intracytoplasmic domain for downstream signaling [66]. Cleaved TLR extracellular domains are not able to mediate downstream signaling, but they participate in regulating TLR signaling and the production of inflammatory mediators as receptor antagonists (decoy receptors) [67]. Thus, we focused on the presence of soluble TLR4 and TLR2 in the intestine, which can indicate the regulation of inflammatory reactions. TLR4 levels were significantly upregulated in both parts of the intestine; however, pre‐colonization with DPM alleviated (in the ileum) or suppressed (in the colon) this increase. The pre‐colonization with DPM suppressed *Salmonella*‐induced TLR2 levels in the colon but not in the ileum. The last observed soluble receptor was CD14, which is also known as “presepsin,” and its serum detection is used as a marker of sepsis and systemic inflammatory response syndrome in newborns [68]. CD14 levels were increased in the ileum but not in the colon of *Salmonella*‐infected piglets.

HMGB1 has the potential to amplify inflammatory reactions [58]. Like LBP, it can concentrate LPS for recognition by TLR4 or TLR2. Moreover, HMGB1 also binds to lipoteichoic acid (LTA), a Gram‐positive bacterial PAMP, which is stated as the primary bacterial ligand for TLR2 and transfers it to CD14 [69].

Pre‐colonization of germ‐free (GF) piglets with a defined porcine microbiota conferred protection against subsequent *Salmonella enterica* serovar Typhimurium LT2 infection over 24 h. Further studies are needed to determine whether this protective effect extends to long‐term resistance against *S*. Typhimurium LT2, which is otherwise lethal in unprotected GF piglets.

## Author Contributions

Alla Splichalova and Igor Splichal conceived and directed the study. Alla Splichalova, Igor Splichal, Katerina Polakova, Marek Sinkora, Vera Neuzil Bunesova, Nikol Modrackova, Eva Vlkova, and Kristyna Horvathova carried out the experiments. Alla Splichalova, Igor Splichal, and Katerina Polakova wrote the manuscript draft. Eva Vlkova supervised the project. Alla Splichalova and Igor Splichal validated the experiment. Alla Splichalova, Igor Splichal, Katerina Polakova, Zdislava Kindlova, and Marek Sinkora were involved in data analyses. Vera Neuzil Bunesova, Nikol Modrackova, Eva Vlkova, Marek Sinkora, Kristyna Horvathova, and Zdislava Kindlova provided critical revision of the manuscript for important intellectual content. All authors read and approved the final manuscript.

## Funding

This work was supported by Czech Science Foundation (21‐15621S) and Ministry of Education, Youth and Sports of the Czech Republic (LM2023064).

## Disclosure

The authors have nothing to report.

## Ethics Statement

The work with animals was conducted in accordance with the ethical standards defined by EU legislation on the use of experimental animals (2010/63/EU) and approved by the Animal Care and Use Committee of the Czech Academy of Sciences (protocol 57/2021, dated August 18, 2021).

## Conflicts of Interest

The authors declare no conflicts of interest.

## Data Availability

All data underlying the results are available in the article, and no additional source data are needed.

## References

[1] K. A. Krautkramer , J. Fan , and F. Bäckhed , “Gut Microbial Metabolites as Multi‐Kingdom Intermediates,” Nature Reviews. Microbiology 19 (2021): 77–94, 10.1038/s41579-020-0438-4. 32968241

[2] M. Fassarella , E. E. Blaak , J. Penders , A. Nauta , H. Smidt , and E. G. Zoetendal , “Gut Microbiome Stability and Resilience: Elucidating the Response to Perturbations in Order to Modulate Gut Health,” Gut 70 (2021): 595–605, 10.1136/gutjnl-2020-321747. 33051190

[3] K. Jaswal , O. A. Todd , and J. Behnsen , “Neglected Gut Microbiome: Interactions of the Non‐Bacterial Gut Microbiota With Enteric Pathogens,” Gut Microbes 15 (2023): 2226916, 10.1080/19490976.2023.2226916. 37365731 PMC10305517

[4] K. M. Kennedy , M. C. de Goffau , M. E. Perez‐Muñoz , et al., “Questioning the Fetal Microbiome Illustrates Pitfalls of Low‐Biomass Microbial Studies,” Nature 613 (2023): 639–649, 10.1038/s41586-022-05546-8. 36697862 PMC11333990

[5] H. Enav , F. Bäckhed , and R. E. Ley , “The Developing Infant Gut Microbiome: A Strain‐Level View,” Cell Host & Microbe 30 (2022): 627–638, 10.1016/j.chom.2022.04.009. 35550666

[6] M. R. G. Carrapato , A. M. Ferreira , and T. Wataganara , “Cesarean Section: The Pediatricians' Views,” Journal of Maternal‐Fetal & Neonatal Medicine 30 (2017): 2081–2085, 10.1080/14767058.2016.1237496. 27659100

[7] S. A. Adugna , J. D. Kitessa , C. T. Feyissa , and S. A. Adem , “Review on a Cesarean Section in the Cow: Its Incision Approaches, Relative Advantage, and Disadvantages,” Veterinary Medicine and Science 8 (2022): 1626–1631, 10.1002/vms3.808. 35474614 PMC9297780

[8] J. R. Greghi , P. O. Favaron , L. G. C. Trautwein , C. G. B. da Silva , G. A. A. de Lemos , and M. I. M. Martins , “Emergency Cesarean Section in Dogs: Usefulness of Amniotic Fluid Biochemical Parameters and Placental Morphology as Indicators of Neonatal Viability,” Theriogenology 211 (2023): 115–124, 10.1016/j.theriogenology.2023.08.011. 37607467

[9] C. Tirone , L. Pezza , A. Paladini , et al., “Gut and Lung Microbiota in Preterm Infants: Immunological Modulation and Implication in Neonatal Outcomes,” Frontiers in Immunology 10 (2019): 2910, 10.3389/fimmu.2019.02910. 31921169 PMC6920179

[10] D. Marty , K. Sorum , K. Smith , P. Nicoski , B. A. Sayyed , and S. Amin , “Nosocomial Infections in the Neonatal Intensive Care Unit,” NeoReviews 25 (2024): e254–e264, 10.1542/neo.25-5-e254. 38688885

[11] S. A. V. Jennings and T. Clavel , “Synthetic Communities of Gut Microbes for Basic Research and Translational Approaches in Animal Health and Nutrition,” Annual Review of Animal Biosciences 12 (2024): 283–300, 10.1146/annurev-animal-021022-025552. 37963399

[12] G. Caballero‐Flores , J. M. Pickard , and G. Núñez , “Microbiota‐Mediated Colonization Resistance: Mechanisms and Regulation,” Nature Reviews. Microbiology 21 (2023): 347–360, 10.1038/s41579-022-00833-7. 36539611 PMC10249723

[13] J. Han , N. Aljahdali , S. Zhao , et al., “Infection Biology of *Salmonella Enterica* ,” EcoSal Plus 12 (2024): 12023, 10.1128/ecosalplus.esp-0001-2023. PMC11636313 38415623

[14] J. Campos , J. Mourão , L. Peixe , and P. Antunes , “Non‐Typhoidal Salmonella in the Pig Production Chain: A Comprehensive Analysis of Its Impact on Human Health,” Pathogens 8 (2019): 19, 10.3390/pathogens8010019. 30700039 PMC6470815

[15] R. G. Ferrari , D. K. A. Rosario , A. Cunha‐Neto , S. B. Mano , E. E. S. Figueiredo , and C. A. Conte‐Junior , “Worldwide Epidemiology of Salmonella Serovars in Animal‐Based Foods: A Meta‐Analysis,” Applied and Environmental Microbiology 85 (2019): 91‐19, 10.1128/AEM.00591-19. PMC6606869 31053586

[16] K. A. Fitzgerald and J. C. Kagan , “Toll‐Like Receptors and the Control of Immunity,” Cell 180 (2020): 1044–1066, 10.1016/j.cell.2020.02.041. 32164908 PMC9358771

[17] T. Gong , L. Liu , W. Jiang , and R. Zhou , “DAMP‐Sensing Receptors in Sterile Inflammation and Inflammatory Diseases,” Nature Reviews. Immunology 20 (2020): 95–112, 10.1038/s41577-019-0215-7. 31558839

[18] T. Kawai and S. Akira , “Toll‐Like Receptors and Their Crosstalk With Other Innate Receptors in Infection and Immunity,” Immunity 34 (2011): 637–650, 10.1016/j.immuni.2011.05.006. 21616434

[19] M. McClelland , K. E. Sanderson , J. Spieth , et al., “Complete Genome Sequence of Salmonella Enterica Serovar Typhimurium LT2,” Nature 413 (2001): 852–856, 10.1038/35101614. 11677609

[20] R. C. Clarke and C. L. Gyles , “Virulence of Wild and Mutant Strains of *Salmonella Typhimurium* in Ligated Intestinal Segments of Calves, Pigs, and Rabbits,” American Journal of Veterinary Research 48 (1987): 504–510. 3551701

[21] I. Splichal , I. Rychlik , I. Splichalova , D. Karasova , and A. Splichalova , “Toll‐Like Receptor 4 Signaling in the Ileum and Colon of Gnotobiotic Piglets Infected With *Salmonella Typhimurium* or Its Isogenic ∆Rfa Mutants,” Toxins 12 (2020): 545, 10.3390/toxins12090545. 32842482 PMC7551901

[22] K. Horvathova , N. Modrackova , I. Splichal , et al., “Defined Pig Microbiota With a Potential Protective Effect Against Infection With *Salmonella Typhimurium* ,” Microorganisms 11 (2023): 1007, 10.3390/microorganisms11041007. 37110429 PMC10146858

[23] N. Modrackova , K. Horvathova , C. Mekadim , et al., “Defined Pig Microbiota Mixture as Promising Strategy Against Salmonellosis in Gnotobiotic Piglets,” Animals 14 (2024): 1779, 10.3390/ani14121779. 38929398 PMC11200913

[24] I. Trebichavsky , V. Dlabac , Z. Rehakova , M. Zahradnickova , and I. Splichal , “Cellular Changes and Cytokine Expression in the Ilea of Gnotobiotic Piglets Resulting From Peroral *Salmonella Typhimurium* Challenge,” Infection and Immunity 65 (1997): 5244–5249, 10.1128/iai.65.12.5244-5249.1997. 9393822 PMC175755

[25] A. Splichalova , V. Slavikova , Z. Splichalova , and I. Splichal , “Preterm Life in Sterile Conditions: A Study on Preterm, Germ‐Free Piglets,” Frontiers in Immunology 9 (2018): 220, 10.3389/fimmu.2018.00220. 29491864 PMC5817058

[26] J.‐H. Xing , T.‐M. Niu , B.‐S. Zou , et al., “Gut Microbiota‐Derived LCA Mediates the Protective Effect of PEDV Infection in Piglets,” Microbiome 12 (2024): 20, 10.1186/s40168-023-01734-4. 38317217 PMC10840300

[27] I. Splichal , S. M. Donovan , Z. Splichalova , et al., “Colonization of Germ‐Free Piglets With Commensal *Lactobacillus Amylovorus*, *Lactobacillus Mucosae*, and Probiotic *E. Coli* Nissle 1917 and Their Interference With *Salmonella Typhimurium* ,” Microorganisms 7 (2019): 0273, 10.3390/microorganisms7080273. PMC6722580 31434337

[28] A. Splichalova , R. Pechar , J. Killer , et al., “Colonization of Germ‐Free Piglets With Mucinolytic and Non‐Mucinolytic *Bifidobacterium Boum* Strains Isolated From the Intestine of Wild Boar and Their Interference With *Salmonella Typhimurium* ,” Microorganisms 8 (2020): 2002, 10.3390/microorganisms8122002. 33333934 PMC7765441

[29] T. D. Schmittgen and K. J. Livak , “Analyzing Real‐Time PCR Data by the Comparative C(T) Method,” Nature Protocols 3 (2008): 1101–1108, 10.1038/nprot.2008.73. 18546601

[30] R. M. Roberts , J. A. Green , and L. C. Schulz , “The Evolution of the Placenta,” Reproduction 152 (2016): R179–R189, 10.1530/REP-16-0325. 27486265 PMC5033709

[31] N. A. Bigler , R. M. Bruckmaier , and J. J. Gross , “Implications of Placentation Type on Species‐Specific Colostrum Properties in Mammals,” Journal of Animal Science 100 (2022): skac287, 10.1093/jas/skac287. 36048628 PMC9713508

[32] H. Salmon , M. Berri , V. Gerdts , and F. Meurens , “Humoral and Cellular Factors of Maternal Immunity in Swine,” Developmental and Comparative Immunology 33 (2009): 384–393, 10.1016/j.dci.2008.07.007. 18761034

[33] O. P. Miniats and D. Jol , “Gnotobiotic Pigs‐Derivation and Rearing,” Canadian Journal of Comparative Medicine 42 (1978): 428–437. 154359 PMC1277667

[34] O. Bæk , A. Brunse , D. N. Nguyen , A. Moodley , T. Thymann , and P. T. Sangild , “Diet Modulates the High Sensitivity to Systemic Infection in Newborn Preterm Pigs,” Frontiers in Immunology 11 (2020): 1019, 10.3389/fimmu.2020.01019. 32536925 PMC7267211

[35] I. S. Arda , F. Ergin , B. Varan , B. Demirhan , H. Aslan , and I. Ozyaylali , “Acute Abdomen Caused by *Salmonella Typhimurium* Infection in Children,” Journal of Pediatric Surgery 36 (2001): 1849–1852, 10.1053/jpsu.2001.28867. 11733922

[36] T. Skrzypek , J. L. Valverde Piedra , H. Skrzypek , W. Kazimierczak , M. Biernat , and R. Zabielski , “Gradual Disappearance of Vacuolated Enterocytes in the Small Intestine of Neonatal Piglets,” Journal of Physiology and Pharmacology 58, no. 3 (2007): 87–95. 17901585

[37] A. Splichalova , Z. Splichalova , D. Karasova , et al., “Impact of the Lipopolysaccharide Chemotype of *Salmonella Enterica* Serovar Typhimurium on Virulence in Gnotobiotic Piglets,” Toxins 11 (2019): 534, 10.3390/toxins11090534. 31540295 PMC6784012

[38] T. W. Shirkey , R. H. Siggers , B. G. Goldade , et al., “Effects of Commensal Bacteria on Intestinal Morphology and Expression of Proinflammatory Cytokines in the Gnotobiotic Pig,” Experimental Biology and Medicine 231 (2006): 1333–1345, 10.1177/153537020623100807. 16946402

[39] J. M. Allaire , S. M. Crowley , H. T. Law , S.‐Y. Chang , H.‐J. Ko , and B. A. Vallance , “The Intestinal Epithelium: Central Coordinator of Mucosal Immunity,” Trends in Immunology 39 (2018): 677–696, 10.1016/j.it.2018.04.002. 29716793

[40] T. Otani and M. Furuse , “Tight Junction Structure and Function Revisited,” Trends in Cell Biology 30 (2020): 805–817, 10.1016/j.tcb.2020.08.004. 33097373

[41] P. Paone and P. D. Cani , “Mucus Barrier, Mucins and Gut Microbiota: The Expected Slimy Partners?,” Gut 69 (2020): 2232–2243, 10.1136/gutjnl-2020-322260. 32917747 PMC7677487

[42] K. Bergstrom , X. Shan , D. Casero , et al., “Proximal Colon‐Derived O‐Glycosylated Mucus Encapsulates and Modulates the Microbiota,” Science 370 (2020): 467–472, 10.1126/science.aay7367. 33093110 PMC8132455

[43] S. K. Linden , P. Sutton , N. G. Karlsson , V. Korolik , and M. A. McGuckin , “Mucins in the Mucosal Barrier to Infection,” Mucosal Immunology 1 (2008): 183–197, 10.1038/mi.2008.5. 19079178 PMC7100821

[44] N. Lhocine , E. T. Arena , P. Bomme , et al., “Apical Invasion of Intestinal Epithelial Cells by *Salmonella Typhimurium* Requires Villin to Remodel the Brush Border Actin Cytoskeleton,” Cell Host & Microbe 17 (2015): 164–177, 10.1016/j.chom.2014.12.003. 25600187 PMC4346658

[45] A. N. Vlasova , F. C. Paim , S. Kandasamy , et al., “Protein Malnutrition Modifies Innate Immunity and Gene Expression by Intestinal Epithelial Cells and Human Rotavirus Infection in Neonatal Gnotobiotic Pigs,” mSphere 2 (2017): 46‐17, 10.1128/mSphere.00046-17. PMC5332602 28261667

[46] S. C. Wen , E. Best , and C. Nourse , “Non‐Typhoidal Salmonella Infections in Children: Review of Literature and Recommendations for Management,” Journal of Paediatrics and Child Health 53 (2017): 936–941, 10.1111/jpc.13585. 28556448

[47] D. Günzel and M. Fromm , “Claudins and Other Tight Junction Proteins,” Comprehensive Physiology 2 (2012): 1819–1852, 10.1002/cphy.c110045. 23723025

[48] N. Bellido‐Carreras , H. Argüello , S. Zaldívar‐López , et al., “ *Salmonella Typhimurium* Infection Along the Porcine Gastrointestinal Tract and Associated Lymphoid Tissues,” Veterinary Pathology 56 (2019): 681–690, 10.1177/0300985819843682. 31106677

[49] R. Al‐Sadi , K. Khatib , S. Guo , D. Ye , M. Youssef , and T. Ma , “Occludin Regulates Macromolecule Flux Across the Intestinal Epithelial Tight Junction Barrier,” American Journal of Physiology. Gastrointestinal and Liver Physiology 300 (2011): G1054–G1064, 10.1152/ajpgi.00055.2011. 21415414 PMC3119114

[50] A. Splichalova , I. Trebichavsky , V. Rada , E. Vlkova , U. Sonnenborn , and I. Splichal , “Interference of *Bifidobacterium Choerinum* or *Escherichia Coli* Nissle 1917 With *Salmonella Typhimurium* in Gnotobiotic Piglets Correlates With Cytokine Patterns in Blood and Intestine,” Clinical and Experimental Immunology 163 (2011): 242–249, 10.1111/j.1365-2249.2010.04283.x. 21155989 PMC3043315

[51] J. C. Kagan , “Lipopolysaccharide Detection Across the Kingdoms of Life,” Trends in Immunology 38 (2017): 696–704, 10.1016/j.it.2017.05.001. 28551077 PMC5624813

[52] D. A. Kaufman , P. H. Perks , R. G. Greenberg , and D. Jensen , “Endotoxin Content in Neonatal Formulas, Fortification, and Lactoferrin Products: Association With Outcomes and Guidance on Acceptable Limits,” Biometals 36 (2023): 703–708, 10.1007/s10534-022-00487-1. 36705875 PMC10181959

[53] S. Kumar , V. Sharma , and S. Yadav , “TLR4 Targeting: A Promising Therapeutic Approach Across Multiple Human Diseases,” Current Protein & Peptide Science 26 (2025): 241–258, 10.2174/0113892037324425241018061548. 39722483

[54] H. Potockova , J. Sinkorova , K. Karova , and M. Sinkora , “The Distribution of Lymphoid Cells in the Small Intestine of Germ‐Free and Conventional Piglets,” Developmental and Comparative Immunology 51 (2015): 99–107, 10.1016/j.dci.2015.02.014. 25743381

[55] R. Pabst , “The Pig as a Model for Immunology Research,” Cell and Tissue Research 380 (2020): 287–304, 10.1007/s00441-020-03206-9. 32356014 PMC7223737

[56] D. C. Fajgenbaum and C. H. June , “Cytokine Storm,” New England Journal of Medicine 383 (2020): 2255–2273, 10.1056/NEJMra2026131. 33264547 PMC7727315

[57] H. Matsumoto , H. Ogura , K. Shimizu , et al., “The Clinical Importance of a Cytokine Network in the Acute Phase of Sepsis,” Scientific Reports 8 (2018): 13995, 10.1038/s41598-018-32275-8. 30228372 PMC6143513

[58] U. Andersson and H. Yang , “HMGB1 Is a Critical Molecule in the Pathogenesis of Gram‐Negative Sepsis,” Journal of Intensive Medicine 2 (2022): 156–166, 10.1016/j.jointm.2022.02.001. 36789020 PMC9924014

[59] E. B. Thorgersen , B. C. Hellerud , E. W. Nielsen , et al., “CD14 Inhibition Efficiently Attenuates Early Inflammatory and Hemostatic Responses in *Escherichia Coli* Sepsis in Pigs,” FASEB Journal 24 (2010): 712–722, 10.1096/fj.09-140798. 19841036 PMC2830134

[60] L. V. Hooper , D. R. Littman , and A. J. Macpherson , “Interactions Between the Microbiota and the Immune System,” Science 336 (2012): 1268–1273, 10.1126/science.1223490. 22674334 PMC4420145

[61] K. Matsushima , D. Yang , and J. J. Oppenheim , “Interleukin‐8: An Evolving Chemokine,” Cytokine 153 (2022): 155828, 10.1016/j.cyto.2022.155828. 35247648

[62] N. Foster , M. A. Lovell , K. L. Marston , et al., “Rapid Protection of Gnotobiotic Pigs Against Experimental Salmonellosis Following Induction of Polymorphonuclear Leukocytes by Avirulent *Salmonella Enterica* ,” Infection and Immunity 71 (2003): 2182–2191, 10.1128/IAI.71.4.2182-2191.2003. 12654840 PMC152035

[63] I. Splichal , I. Trebichavsky , A. Splichalova , and P. A. Barrow , “Protection of Gnotobiotic Pigs Against *Salmonella Enterica* Serotype Typhimurium by Rough Mutant of the Same Serotype Is Accompanied by the Change of Local and Systemic Cytokine Response,” Veterinary Immunology and Immunopathology 103 (2005): 155–161, 10.1016/j.vetimm.2004.09.001. 15621302

[64] A. Splichalova , I. Splichal , P. Chmelarova , and I. Trebichavsky , “Alarmin HMGB1 Is Released in the Small Intestine of Gnotobiotic Piglets Infected With Enteric Pathogens and Its Level in Plasma Reflects Severity of Sepsis,” Journal of Clinical Immunology 31 (2011): 488–497, 10.1007/s10875-010-9505-3. 21225449

[65] R. Vitali , G. Terrin , F. Palone , et al., “Fecal High‐Mobility Group Box 1 as a Marker of Early Stage of Necrotizing Enterocolitis in Preterm Neonates,” Frontiers in Pediatrics 9 (2021): 672131, 10.3389/fped.2021.672131. 34178888 PMC8222523

[66] A. Stierschneider and C. Wiesner , “Shedding Light on the Molecular and Regulatory Mechanisms of TLR4 Signaling in Endothelial Cells Under Physiological and Inflamed Conditions,” Frontiers in Immunology 14 (2023): 1264889, 10.3389/fimmu.2023.1264889. 38077393 PMC10704247

[67] A.‐C. Raby , E. Le Bouder , C. Colmont , et al., “Soluble TLR2 Reduces Inflammation Without Compromising Bacterial Clearance by Disrupting TLR2 Triggering,” Journal of Immunology (Baltimore, Md.: 1950) 183 (2009): 506–517, 10.4049/jimmunol.0802909. 19542461

[68] M. Mussap , E. Puxeddu , M. Puddu , et al., “Soluble CD14 Subtype (SCD14‐ST) Presepsin in Premature and Full Term Critically Ill Newborns With Sepsis and SIRS,” Clinica Chimica Acta 451 (2015): 65–70, 10.1016/j.cca.2015.07.025. 26232159

[69] M. S. Kwak , M. Lim , Y. J. Lee , et al., “HMGB1 Binds to Lipoteichoic Acid and Enhances TNF‐*α* and IL‐6 Production Through HMGB1‐Mediated Transfer of Lipoteichoic Acid to CD14 and TLR2,” Journal of Innate Immunity 7 (2015): 405–416, 10.1159/000369972. 25660311 PMC6738877

